# Ryk regulates Wnt5a repulsion of mouse corticospinal tract through modulating planar cell polarity signaling

**DOI:** 10.1038/celldisc.2017.15

**Published:** 2017-05-16

**Authors:** Xin Duan, Yarong Gao, Yaobo Liu

**Affiliations:** 1 Institute of Neuroscience, Jiangsu Key Laboratory of Translational Research and Therapy for Neuro-Psycho-Diseases, Soochow University, Suzhou, China

**Keywords:** Ryk, Wnt5a, repulsion, planar cell polarity signaling, corticospinal tract

## Abstract

It was previously reported a role for Ryk in mediating Wnt5a repulsion of the corticospinal tract (CST) in mice. Recent evidence has shown that Ryk regulates planar cell polarity (PCP) signaling through interacting with Vangl2. Here, *in vivo*, *in vitro* and biochemical analyses were applied to investigate the molecular cross-talk between the Ryk and PCP signaling pathways, revealing that PCP pathway components play important roles in CST anterior–posterior guidance. Ryk–Vangl2 interactions are crucial for PCP signaling to mediate Wnt5a repulsion of CST axons. Cytoplasmic distribution of Ryk is increased under high concentrations of Wnt5a and facilitates the cytoplasmic distribution of Vangl2, leading to inhibition of Frizzled3 translocation to cytoplasm. Alternatively, Ryk stabilizes Vangl2 in the plasma membrane under low Wnt5a concentrations, which promotes cytoplasmic translocation of Frizzled3. We propose that Ryk regulates PCP signaling through asymmetric modulation of Vangl2 distribution in the cytoplasm and plasma membrane, which leads to repulsion of CST axons in response to the Wnt gradient.

## Introduction

In the mammalian central nervous system (CNS), the corticospinal tract (CST) is the major motor pathway contributing to the control of voluntary movements and motor functions of the body [1]. It forms the longest axonal trajectory in the central nervous system, and its axons navigate the entire length of the central nervous system. CST axons first project from the frontal and sensorimotor cortex, and then travel through the cerebral peduncles and brain stem. The majority of these axons cross over to the opposite side of the brain stem and enter the dorsal funiculus. These axons travel down the tract in the white matter of the spinal cord until they reach the proper position [2–4]. This period of navigation is extremely complex and relies on the coordinated regulation of guidance cues such as ephrins [5], netrins [6, 7], semaphorins [8], slits [9, 10] and Wnts [11, 12].

Following the discovery that Wnt proteins can attract axons up the spinal cord toward the brain via Frizzled3 (Fzd3) [13], previous studies also found that mouse CST axons are repelled by the high Wnt5a expression in the rostral spinal cord relative to the low expression in caudal spinal cord, and this repulsion is mediated by the receptor tyrosine kinase Ryk [12]. Ryk acts as a Wnt receptor and has been shown to function in several processes, including axon repulsion, axon extension and neuronal differentiation [12, 14–16].

Recent studies have begun to reveal the role of the planar cell polarity (PCP) signaling pathway in axon guidance and the potential signaling mechanisms involved in growth cone guidance [17]. Core PCP pathway components, such as the four-pass transmembrane protein Vangl2, the Wnt-binding cell surface receptor Fzd3 and the downstream signal mediator disheveled (Dvl), have been found to be required for attractive anterior–posterior (A–P) guidance of brain stem serotonergic and dopaminergic axons and spinal cord commissural axons [18–20]. Moreover, both Ryk and Fzd3 receptors seem to be involved in Wnt repulsion of the corpus callosum *in vitro* [15]. These studies support the view that the PCP signaling pathway may provide a major axon steering mechanism in response to Wnts and may be a commonly used pathway for bidirectional control of axon guidance in the A–P axis. Furthermore, Ryk has been reported to interact with Vangl2 genetically and biochemically, and the interaction is enhanced by Wnt5a. Mechanistically, Ryk regulates the PCP pathway by binding to Vangl2 and increasing its stability [21]. These findings strongly indicate that Ryk may mediate Wnt repulsion of axons through modulating PCP signaling. However, the mechanisms underlying this modulation remain unknown.

Here, we present evidence suggesting that, in murine growth cones of corticospinal axons responding to the Wnt5a gradient, increased cytoplasmic distribution of Vangl2 occurs predominantly toward higher Wnt5a concentration, apparently mediated through Ryk–Vangl2 interactions and translocation of Ryk to the cytoplasm, whereas Vangl2 is retained in the cell membrane on the side of lower Wnt5a concentration through Ryk–Vangl2 interactions. The asymmetric distribution of Vangl2 results in the amplification of PCP signaling on the side of lower Wnt5a concentration and corresponding growth cone turning toward the lower Wnt5a concentration, which further leads to repulsive behavior of the corticospinal axonal growth cone in response to the Wnt5a gradient.

## Results

### Expression of PCP components is upregulated in neonatal corticospinal neurons and axons

**Figure 1:**
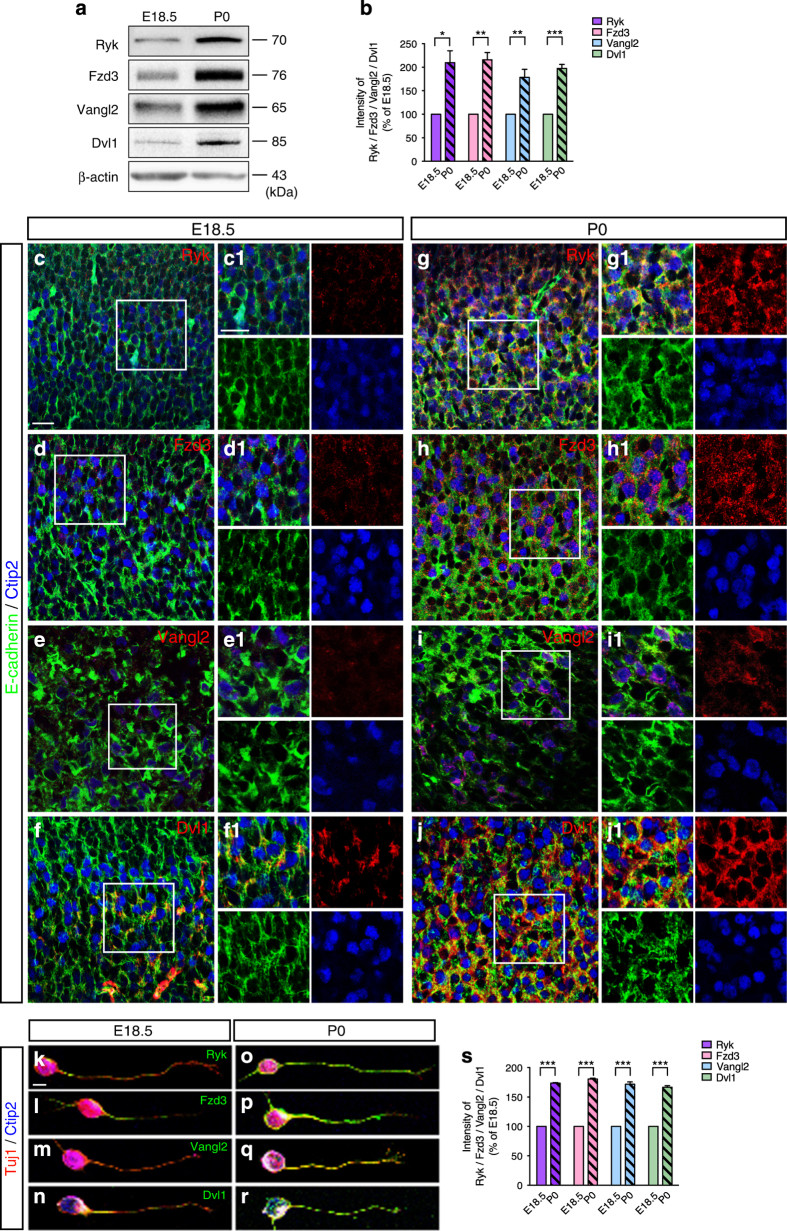
Upregulation of Ryk, Fzd3, Vangl2 and Dvl1 in developing corticospinal neurons and axons. (**a**) Western blot analysis of Ryk and core PCP pathway components Fzd3, Vangl2 and Dvl1 in E18.5 and P0 cortex. (**b**) Quantification of Ryk, Fzd3, Vangl2 and Dvl1 immunoblotting intensity in E18.5 and P0 cortex. Data are represented as the mean±s.e.m. *P<0.05, **P<0.01, ***P<0.001 (100% of E18.5, P0 Ryk=210±25.3%, *t*=4.34, *P*=0.0123; P0 Fzd3=216±15.4%, *t*=7.53, *P*=0.0017; P0 Vangl2=178±17.0%, *t*=4.63, *P*=0.0098; P0 Dvl1=197±8.36%, *t*=11.7, *P*=0.0003. Student’s *t*-test). Data were analyzed from three independent experiments, each including three mice per group. (**c**–**f**) Expression of Ryk (**c**) and core PCP pathway components Fzd3 (**d**), Vangl2 (**e**) and Dvl1 (**f**) in E18.5 corticospinal neurons. (**g**–**j**) Expression of Ryk (**g**) and core PCP pathway components Fzd3 (**h**), Vangl2 (**i**) and Dvl1 (**j**) in P0 corticospinal neurons. Co-staining was carried out with Ctip2 (blue) and E-cadherin (green) antibodies and Ryk, Fzd3, Vangl2 or Dvl1 antibodies (red). Scale bar, 50 μm. (**c1**–**f1**) Higher magnification images of the boxed areas in **c**–**f**. Scale bar, 10 μm. (**k**–**n**) Expression of Ryk (**k**) and core PCP pathway components Fzd3 (**l**), Vangl2 (**m**) and Dvl1 (**n**) in E18.5 corticospinal axons. (**o**–**r**) Expression of Ryk (**o**) and core PCP pathway components Fzd3 (**p**), Vangl2 (**q**) and Dvl1 (**r**) in P0 corticospinal axons. Co-staining was carried out with Ctip2 (blue), Tuj1 (red) antibodies and Ryk, Fzd3, Vangl2 or Dvl1 antibodies (green). Scale bar, 5 μm. (**s**) Quantification of Ryk, Fzd3, Vangl2 and Dvl1 staining intensity in E18.5 and P0 corticospinal neurons. Data are represented as the mean±s.e.m. ***P<0.001 (100% of E18.5, P0 Ryk=174±0.83%, *t*=88.5; P0 Fzd3=181±1.37%, *t*=58.8; P0 Vangl2=172±3.99%, *t*=18.0; P0 Dvl1=167±2.66%, *t*=25.0. Student’s *t*-test). Data were analyzed from three independent experiments, each including 20 corticospinal neurons per group.

Previous work showed that Ryk is expressed in layer 5 of the frontal and sensorimotor cortex at P0 (postnatal day 0) in mice, but is barely detectable in layer 5 at E18.5 (embryonic day 18.5) [12]. To investigate whether the Wnt/PCP signaling pathway is involved in A–P guidance of corticospinal axons, we first detected the expression of the core PCP pathway components in the developing mouse cortex using western blotting. We used Ryk as a positive control to examine the expression of Fzd3, Vangl2 and Dvl1. The obvious upregulation of Ryk, Fzd3, Vangl2 and Dvl1 proteins was detected in P0 mouse cortex (Figure 1a). The quantification showed that the expression of Ryk, Fzd3, Vangl2 and Dvl1 proteins in P0 cortex was around twofold higher than in E18.5 cortex (Figure 1b). We further analyzed the expression patterns of the core PCP pathway components in the developing corticospinal neurons of mice using immunostaining. Anti-Ctip2 staining was used as the marker of neurons in layer 5 and anti-E-cadherin staining was used to show the cell membrane. Ryk, Fzd3, Vangl2 and Dvl1 were significantly and specifically expressed in Ctip2-positive neurons of layer 5 at P0, whereas their expression was relatively weak at E18.5 (Figure 1c–j). The obvious upregulation of Ryk, Fzd3, Vangl2 and Dvl1 was observed in the somas of P0 corticospinal neurons (Figure 1c1–j1).

To further examine the expression patterns of Ryk, Fzd3, Vangl2 and Dvl1 in corticospinal axons, we dissociated neurons of cerebral cortex layer 5. Ctip2 and Tuj1 antibodies were used as markers to specifically identify the somas and axons of corticospinal neurons, respectively. In addition to the upregulated expression of Ryk, Fzd3, Vangl2 and Dvl1 in the somas of P0 corticospinal neurons, the expression of Ryk, Fzd3, Vangl2 and Dvl1 also significantly increased in P0 corticospinal axons compared with E18.5 corticospinal axons (Figure 1k–r). We separately measured the intensity of Ryk, Fzd3, Vangl2 and Dvl1 expression in the somas and axons, and the quantification showed that the expression of Ryk, Fzd3, Vangl2 and Dvl1 in P0 corticospinal neurons was nearly twofold higher than in E18.5 corticospinal neurons (Figure 1s) and all the upregulations were significant. The specificity for the antibodies of Ryk, Fzd3, Vangl2 and Dvl1 were confirmed using immunostaining of HEK293T cells transfected either with Ryk-pcDNA3.1, Fzd3-pCAGEN, Vangl2-pCMV and Dvl1-pCMV, respectively, or with pcDNA3.1, pCAGEN and pCMV as control (Supplementary Figure S1a–d).

Taken together, these findings show that PCP pathway components are significantly upregulated in the somas and axons of P0 corticospinal neurons when corticospinal axons initiate descent in the spinal cord [22]. Thus, these components of the PCP signaling pathway seem to be potential regulators of A–P guidance of corticospinal axons in the developing spinal cord.

### PCP components mediate Wnt5a repulsion of corticospinal axon growth cones

**Figure 2:**
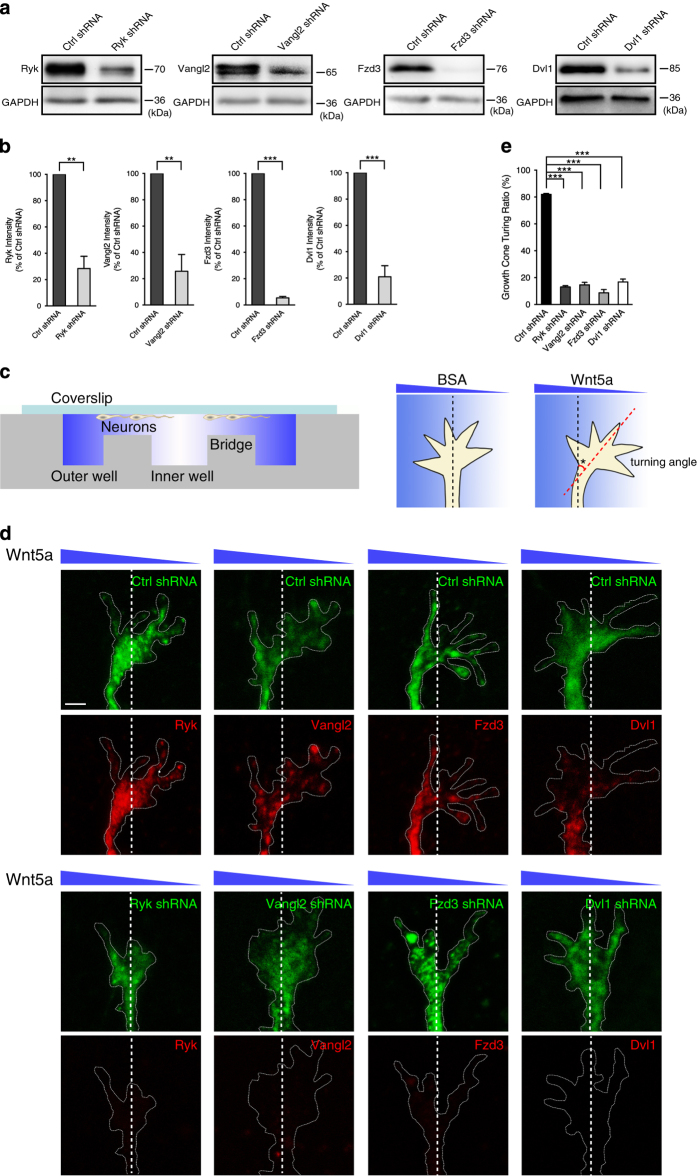
PCP pathway components mediate Wnt5a repulsion of corticospinal axon growth cones. (**a**) Analysis of the knockdown efficiency of Ryk, Vangl2, Fzd3 and Dvl1 shRNA by western blotting. GAPDH was used as an internal control. (**b**) Quantification of the knockdown efficiency of Ryk, Vangl2, Fzd3 and Dvl1 shRNA. Data are represented as the mean±s.e.m. **P<0.01, ***P<0.001 (100% of control shRNA, Ryk shRNA=28.4±9.3%, *t*=7.70, *P*=0.0015; Vangl2 shRNA=25.6±12.8%, *t*=5.82, *P*=0.0011; Fzd3 shRNA=5.3±1.1%, *t*=84.30, *P*<0.0001; Dvl1 shRNA=21.0±8.4%, *t*=9.40. Student’s *t*-test). Data were analyzed from three independent experiments per group. (**c**) Schematic diagram of *in vivo* experimental design to expose corticospinal axon growth cones to Wnt5a gradient using the Dunn chamber. Asterisk indicates the turning angel between midline (black dotted line) and the final position of the growth cone (red dotted line). (**d**) Growth cones of corticospinal axons expressing control shRNA or Ryk, Vangl2, Fzd3 or Dvl1 shRNA responding to Wnt5a gradient. Lentivirus-infected growth cones were identified with EGFP expression (green), and immunostaining was carried out with Ryk, Vangl2, Fzd3 or Dvl1 antibodies (red). Dashed lines outline the growth cones and indicate the midline of the growth cones. Scale bar, 2 μm. (**e**) Quantification of growth cone-turning ratio in the different groups. Data are represented as the mean±s.e.m. ***P<0.001 (control shRNA=82.0±0.67%, Ryk shRNA=13.0±1.03%, Vangl2 shRNA=14.6±1.89%, Fzd3 shRNA=8.6±2.46%, Dvl1 shRNA=16.7±2.21%. One-way ANOVA). Data were analyzed from three independent experiments. Each independent experiment included at least 18 neurons per group.

During the development of the nervous system, corticospinal axons project along the A–P axis in the spinal cord. Wnt5a is thought to repel corticospinal axons during this process and the repulsion is mediated by Ryk [12]. To test whether PCP pathway components are involved in A–P guidance of corticospinal axons, we designed and packaged short hairpin RNA (shRNA)-expressing lentivirus targeting Ryk, Vangl2, Fzd3 or Dvl1 for *in vitro* infection studies. First, we evaluated the knockdown efficiency of shRNA in corticospinal neurons by western blotting. All of the shRNA-expressing lentiviruses strongly downregulated the expression of their respective target (Figure 2a, quantified in Figure 2b). To test whether PCP pathway components mediate corticospinal axon growth cones turning, primary corticospinal neurons were cultured under a Wnt5a gradient in a Dunn chamber (Figure 2c) [19]. Growth cones of corticospinal axons were infected with each of the different shRNA lentiviruses and exposed to the Wnt5a gradient using the Dunn chamber. Thirty minutes after the addition of Wnt5a gradient, corticospinal neurons were fixed. Thus, the growth cones of corticospinal axons would have been in a stable gradient for 10 min [19]. Immunostaining of Ryk, Vangl2, Fzd3 and Dvl1 was carried out to confirm their downregulated expression in the shRNA-expressing growth cones. In negative control-shRNA-expressing neurons, higher concentration of Wnt5a significantly repelled growth cone outgrowth and growth cones were shifted toward the lower concentration of Wnt5a (Figure 2d). In contrast, this growth cone turning to low concentration of Wnt5a was efficiently abolished when Ryk, Vangl2, Fzd3 or Dvl1 was knocked down with shRNA (Figure 2d, quantified in Figure 2e). Previous study demonstrated that Ryk mediates the Wnt5a repulsion of corticospinal axons [12]. The observation that knockdown of core components of the PCP signaling pathway could obviously interrupt the Wnt5a repulsion of corticospinal axon growth cone turning like knockdown of Ryk strongly suggested that the PCP signaling pathway plays a role in Ryk-mediated Wnt5a repulsion of corticospinal axons.

### *In vivo* knockdown of PCP components results in A–P guidance defects of corticospinal axons

**Figure 3:**
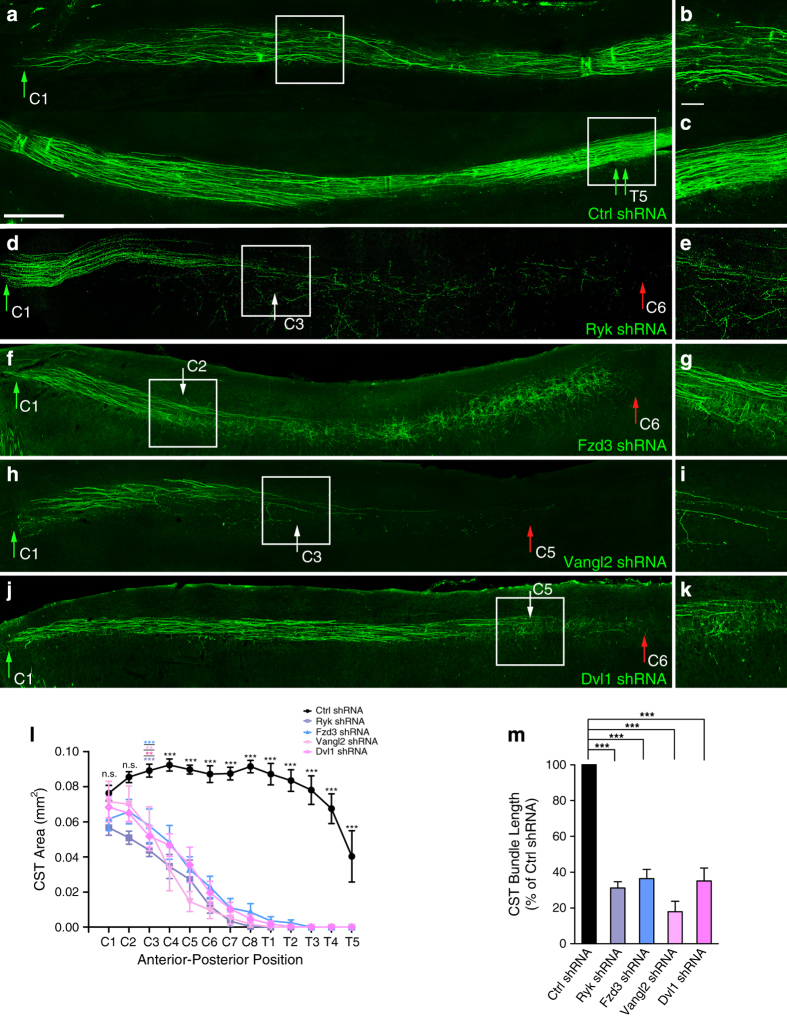
*In vivo* knockdown of Ryk and PCP pathway components results in A–P guidance defects of corticospinal axons. (**a**) Control-shRNA-expressing CST in developing spinal cord. The single green arrow indicates a CST projection initiation site in the spinal cord at first cervical (C1) segment. The double green arrows indicate control-shRNA-expressing CST continuing to descend at the fifth thoracic (T5) segment. (**b**, **c**) Higher magnification images of the boxed areas in **a**. (**d**) Ryk shRNA-expressing CST in developing spinal cord. The white arrow indicates where Ryk shRNA-expressing CST axons initiated randomized extension at the third cervical (C3) segment. The red arrow indicates where Ryk shRNA-expressing CST aberrantly ends at the sixth cervical (C6) segment. (**e**) Higher magnification image of boxed area in **d**. (**f**) Fzd3 shRNA-expressing CST in developing spinal cord. The white arrow indicates where Fzd3 shRNA-expressing CST axons initiated randomized extension at the second cervical (C2) segment. The red arrow indicates where Fzd3 shRNA-expressing CST aberrantly ends at the sixth cervical (C6) segment. (**g**) Higher magnification image of boxed area in **f**. (**h**) Vangl2 shRNA-expressing CST in developing spinal cord. The white arrow indicates where Vangl2 shRNA-expressing CST axons initiated randomized extension at the third cervical (C3) segment. The red arrow indicates where Vangl2 shRNA-expressing CST aberrantly ends at the fifth cervical (C5) segment. (**i**) Higher magnification image of the boxed area in **h**. (**j**) Dvl1 shRNA-expressing CST in developing spinal cord. The white arrow indicates where Dvl1 shRNA-expressing CST axons initiated randomized extension at the fifth cervical (C5) segment. The red arrow indicates where Dvl1 shRNA-expressing CST aberrantly ends at the sixth cervical (C6) segment. (**k**) Higher magnification image of the boxed area in **j**. Axons were identified with EGFP expression (green). Scale bar, 500 or 100 μm (higher magnification images). (**l**, **m**) Quantification of CST area and CST bundle length in each shRNA-expressing group. Data are represented as the mean±s.e.m. **P<0.01, ***P<0.001. Data of CST area and CST bundle length were analyzed from at least seven mice in each group using two-way ANOVA (**l**) or one-way ANOVA (**m**, 100% of control shRNA, Ryk shRNA=31.14±3.526%; Fzd3 shRNA=36.37±7.392%; Vangl2 shRNA=17.95±7.634%; Dvl1 shRNA=35.11±7.635%; *P*<0.001), respectively. NS, not significant.

Due to the importance of Fzd3, Vangl2 and Dvl1 in many earlier developmental processes prior to CST-axon pathfinding, knockdown of the expression of PCP pathway components was applied to analyze whether PCP signaling is required for A–P guidance of corticospinal axons *in vivo*. We injected Fzd3, Vangl2 or Dvl1 shRNA-expressing lentivirus into corticospinal neurons of P0 mice. And injection of Ryk shRNA-expressing lentivirus was used as a positive control following previous report [12]. These mice were then killed 2 weeks post injection and sagittal sections of spinal cord were taken for analysis. The control-shRNA-expressing CST axons continued to extend over the fifth thoracic (T5) segment at P14 (Figure 3a, double green arrow) and presented normal pathfinding in the entire spinal cord (Figure 3b and c). However, Ryk shRNA-expressing CST axons presented random extension at the third cervical (C3) segment (Figure 3d and e, white arrow in Figure 3d) and aberrantly terminated at the sixth cervical (C6) segment (red arrow in Figure 3d). Fzd3 shRNA-expressing CST axons presented random extension at the second cervical (C2) segment (Figure 3f and g, white arrow in Figure 3f) and aberrantly terminated at the sixth cervical (C6) segment (red arrow in Figure 3f). Vangl2 shRNA-expressing CST axons presented random extension at the third cervical (C3) segment (Figure 3h and i, white arrow in Figure 3h) and aberrantly terminated at the fifth cervical (C5) segment (red arrow in Figure 3h). Dvl1 shRNA-expressing CST axons presented random extension at the fifth cervical (C5) segment (Figure 3j and k, white arrow in Figure 3j) and aberrantly terminated at the sixth cervical (C6) segment (red arrow in Figure 3j). Quantification also showed that the area (Figure 3l) and length (Figure 3m) of Ryk, Fzd3, Vangl2 or Dvl1 shRNA-expressing CST axons descending in the spinal cord were substantially reduced when compared with the control-shRNA-expressing CST axons.

According to the previous report, we have known that in human cells and during *Xenopus* embryogenesis, Wnt/Fz signaling can activate the small GTPase Rho. And Wnt/Fz activation of Rho requires the cytoplasmic protein Dvl [23]. We also know that a downstream effector of PCP pathway, JNK, can be activated by Rho A during convergent extension movement in *Xenopus* [24]. Moreover, JNK, as a PCP downstream effector, can be activated by Wnt5a in commissural axons and is required for the A–P guidance [25]. So, here we overexpressed Rho A in corticospinal neurons of P0 mice that can activate JNK, to rescue the misguided axons and ensure the specific knockdown of PCP pathway components. Cortical injection of the Rho A-overexpressing lentivirus was applied to rescue the projection of Ryk, Fzd3, Vangl2 or Dvl1 shRNA-expressing CST axons. The diagram showed the location of cortical injection (Supplementary Figure S2a). These mice were then killed 2 weeks post injection, and coronal sections of cortex or sagittal sections of spinal cord were taken for analysis. The coronal sections of cortex showed the control EGFP-expressing and Cherry-expressing lentivirus CST neurons (Supplementary Figure S2b,b1,b2), and Ryk, Fzd3, Vangl2 or Dvl1 shRNA-expressing with Rho A-overexpressing CST neurons (Supplementary Figure S2c,c1,c2,d,d1,d2,e,e1,e2,f,f1,f2). The sagittal sections of spinal cord showed the control EGFP-expressing and Cherry-expressing lentivirus CST axons continued to extend over the fifth thoracic (T5) segment at P14 (Supplementary Figure S2g) and presented normal pathfinding in the entire spinal cord (Supplementary Figure S2h and i). And CST axons with cortical co-injection of Ryk, Fzd3, Vangl2 or Dvl1 shRNA-expressing and Rho A-overexpressing lentivirus continued to extend over the second thoracic (T2) segment, third thoracic (T3) segment, fourth thoracic (T4) segment or third thoracic (T3) segment separately at P14 (Supplementary Figure S2j,l,n and p), and presented normal pathfinding in the spinal cord (Supplementary Figure S2k,m,o and q). The quantification of CST bundle length showed there is no significance between control EGFP-expressing CST axons with co-injection of Cherry-expressing lentivirus and Ryk, Fzd3, Vangl2 or Dvl1 shRNA-expressing CST axons with co-injection of Rho A-overexpressing lentivirus (Supplementary Figure S2r). These *in vivo* findings demonstrated that PCP pathway components are required in the A–P pathfinding of CST axons in the spinal cord.

### Ryk and Vangl2 interact in corticospinal neurons

Although the above evidence strongly indicated that the PCP signaling pathway is necessary for Ryk-mediated Wnt5a repulsion of CST axons, the mechanism underlying the cross-talk between Ryk and the PCP signaling pathway was unknown. Ryk is a cell membrane protein with homology to the receptor tyrosine kinase family. Ryk contains an extracellular domain, a transmembrane domain and an intracellular domain, and the intracellular domain is unique among the receptor tyrosine kinase family members [26]. The Ryk extracellular domain includes a Wnt inhibitory factor motif that is believed to be required for Wnt binding [27]. Vangl2 is one of the core components of the PCP signaling pathway and plays an important role in the activation of Wnt/PCP signaling [28, 29]. Recent studies have shown that Ryk can interact with Vangl2 and form a Wnt5a receptor complex [21, 30]. These findings indicate that the interactions of Ryk and Vangl2 might play an important role in the cross-talk between Ryk and the PCP signaling pathway.

**Figure 4:**
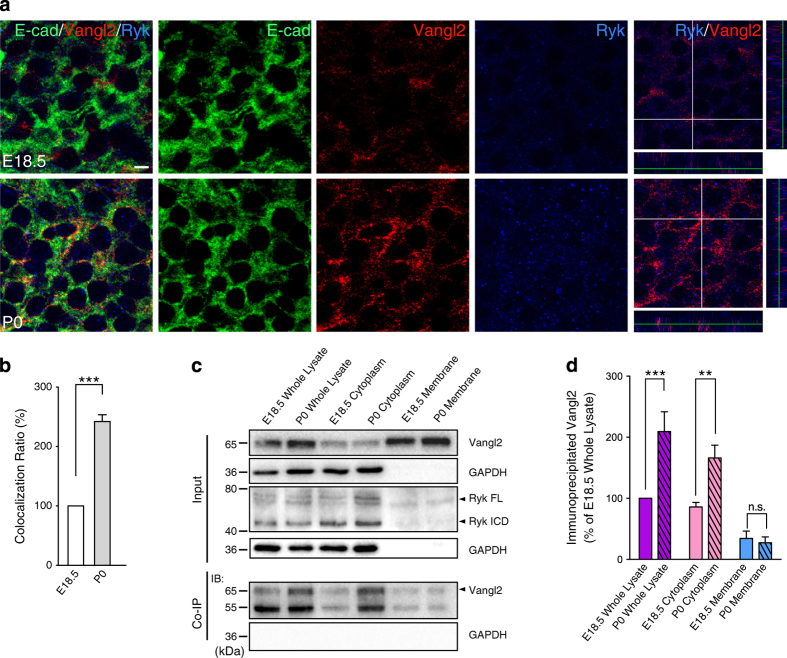
Ryk and Vangl2 interact in corticospinal neurons. (**a**) Co-localization of Ryk and Vangl2 in E18.5 and P0 corticospinal neurons. Co-staining with Ryk (blue), Vangl2 (red) and E-cadherin (E-cad, green) antibodies was carried out on sections from E18.5 and P0 cortex. Anti-E-cadherin staining was used to visualize the cell membrane. Scale bar, 5 μm. (**b**) Quantification of the co-localization of Ryk and Vangl2 from (**a**). Data are represented as the mean±s.e.m. of four independent experiments. ***P<0.001 (100% of E18.5 Ryk and Vangl2 co-localization, P0 Ryk and Vangl2 co-localization=242±11.68%, *t*=12.16, *P*=0.0003, Student’s *t*-test). (**c**) Co-immunoprecipitation (Co-IP) of Ryk and Vangl2 expressed in corticospinal neurons. The whole-cell lysate, membrane and cytoplasm extracts from E18.5 and P0 cortex samples were subjected to co-immunoprecipitation followed by western blot. GAPDH was used as an internal control, and confirmed the separation of the cytosolic and membrane fractions. (**d**) Quantification of co-immunoprecipitated Ryk and Vangl2 in whole-cell lysate, cytoplasm and membrane extracts. Data are represented as the mean±s.e.m. of four independent experiments. **P<0.01, ***P<0.001 (100% of E18.5 whole lysate. first and second columns, 100% of E18.5 whole lysate, P0 whole lysate=209.4±32.19%, *t*=4.74; third and fourth columns, E18.5 cytoplasm=85.794±7.685%, P0 cytoplasm=166.193±20.865%, *t*=3.483; fifth and sixth columns; 100% of E18.5 whole lysate, E18.5 membrane=34.324±12.121%, P0 membrane=27.46±9.411%, *t*=0.2974. Student’s *t*-test). Each independent experiment included six embryos per group. NS, not significant.

To determine whether interactions of Ryk and Vangl2 play a role in the regulation of the pathfinding of corticospinal neurons under the Wnt5a gradient, immunostaining of E18.5 and P0 cortex with Ryk and Vangl2 antibodies was carried out. Both levels of expression and co-localization of Ryk and Vangl2 in the somas of corticospinal neurons were greatly elevated in P0 cortex compared with E18.5 cortex (Figure 4a, quantified in Figure 4b). This indicates that the interactions of Ryk and Vangl2 are enhanced when CST axons start to descend along the P0 spinal cord.

We also verified our findings by co-immunoprecipitation of Ryk and Vangl2 in E18.5 and P0 cortex subcellular fractions. Compared with co-immunoprecipitation of Ryk and Vangl2 from E18.5 cortex, enhanced co-immunoprecipitation of Ryk and Vangl2 was detected in both whole lysate and cytoplasmic fractions from P0 cortex (Figure 4c). Quantification of the immunoprecipitated Vangl2 showed that the interaction between Ryk and Vangl2 was enhanced approximately twofold in whole lysate (Figure 4d, first and second columns) and cytoplasmic fractions (Figure 4d, third and fourth columns) from P0 cortex compared with E18.5 cortex. However, the interaction of Ryk and Vangl2 was weakly detected in membrane fractions from either E18.5 or P0 cortex (Figure 4d, fifth and sixth columns). It indicated that Ryk–Vangl2 interactions might be mainly localized in the growth cones of spinal corticospinal axons responding to Wnt5a gradient. These results confirmed that interaction of Ryk and Vangl2 increased in P0 cortex compared with E18.5 cortex. Taken together, we demonstrate that the interaction between Ryk and Vangl2 is important for mediating Wnt/PCP signaling in regulating the pathfinding of corticospinal axons.

### Increased cytoplasmic distribution of Ryk facilitates Vangl2 distribution in cytoplasm

**Figure 5:**
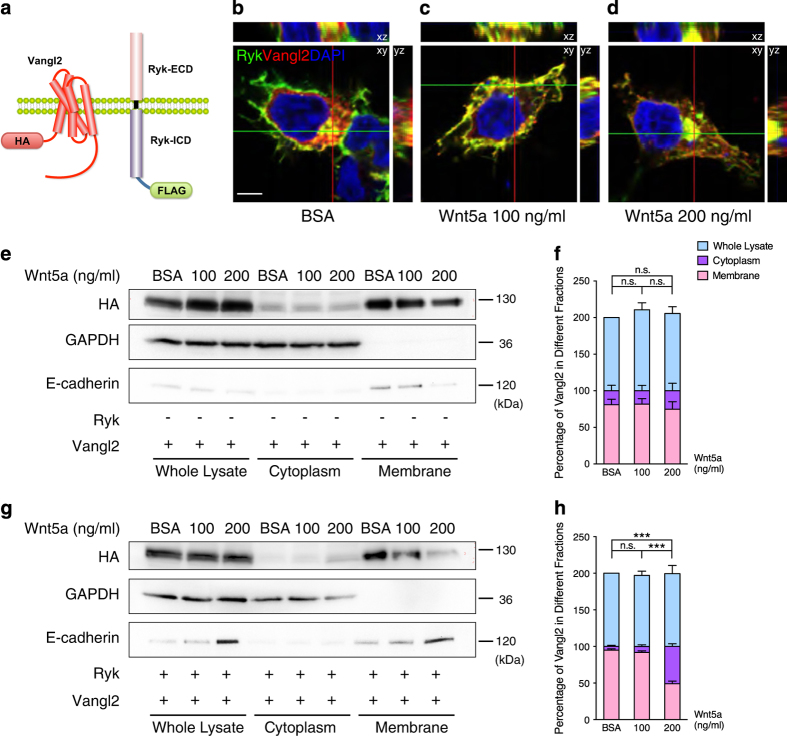
Cytoplasmic translocation of Ryk facilitates cytoplasmic translocation of Vangl2. (**a**) Schematic diagram of Ryk-FLAG and Vanlg2-HA expression constructs. (**b**–**d**) *In vitro* analysis of the distribution of Ryk and Vangl2 in response to Wnt5a gradient. Vangl2-HA and Ryk-FLAG were transfected into HEK293T cells. Vangl2-HA- and Ryk-FLAG-expressing cells were separately treated with BSA (**b**), 100 ng ml^−1^ wnt5a (**c**) or 200 ng ml^−1^ Wnt5a (**d**). DAPI staining (blue) labeled the nucleus. HA (red) and FLAG (green) staining show the expressed Vangl2 and Ryk, respectively. Scale bar, 5 μm. (**e**, **g**) Western blot analysis of whole-cell lysate, cytoplasm and membrane extracts from Vangl2-HA-expressing HEK293T cells with (**g**) or without (**e**) Ryk-FLAG transfection. Antibodies against GAPDH and E-cadherin were used to confirm the purity of the cytoplasm and the membrane fractions, respectively. (**f**, **h**) Quantification of the percentage of Vangl2 in the different fractions in **e**, **g**, respectively (**f**, BSA and 100 ng ml^−1^, *P*=0.9970; BSA and 200 ng ml^−1^, *P*=0.8703; 100 and 200 ng ml^−1^, *P*=0.8345; **h**, BSA and 100 ng ml^−1^, *P*=0.6743). Data are represented as the mean±s.e.m. of at least three independent experiments per group. ***P<0.001, (one-way ANOVA). NS, not significant.

Cleavage of Ryk is known to permit the intracellular C-terminal domain (Ryk ICD) to translocate to the nucleus in response to Wnt stimulation [31], and Ryk transduces Wnt5a signal by forming a complex with Vangl2 [21]. In addition, western blot analysis of subcellular fractions from P0 cortices also showed that the full-length Ryk was localized in the membrane fraction and Ryk ICD localized exclusively in the cytoplasmic fraction (Figure 4c). These results suggest that cytoplasmic distribution of Ryk may be increased with Ryk translocation to the cytoplasm for Ryk-mediated Wnt5a repulsion of corticospinal axons in the spinal cord. To address the questions of how Ryk regulates Vangl2 and the mechanism of signal transduction between Ryk and the PCP signaling pathway, the distribution of Ryk and Vangl2 at the cellular level was examined using a Ryk construct tagged with a FLAG epitope and a Vangl2 construct tagged with an HA epitope (Figure 5a). Different concentrations of Wnt5a were added to HEK293T cells co-transfected with the Ryk-FLAG and Vangl2-HA constructs to mimic the Wnt5a gradient [19]. In the BSA control group, Ryk and Vangl2 were co-localized in both the membrane and cytoplasm (Figure 5b). In the presence of 100 ng ml^−1^ Wnt5a, co-localization of Ryk and Vangl2 strongly increased in the cell membrane (Figure 5c). In the presence of 200 ng ml^−1^ Wnt5a, Ryk and Vangl2 were translocated to the cytoplasm, with strong co-localization (Figure 5d). These results indicated that the interactions of Ryk and Vangl2 were enhanced by either high or low concentrations of Wnt5a. However, Ryk and Vangl2 were maintained in the membrane under low concentration of Wnt5a and translocated to the cytoplasm under high concentration of Wnt5a.

Western blot analysis of subcellular fractions showed that when only Vangl2 was expressed in cells, there was no detectable change in the localization of Vangl2 with increasing concentration of Wnt5a (Figure 5e, quantified in Figure 5f). This suggested that Vangl2 is localized in the membrane and this localization does not change in response to increasing Wnt5a. However, when Ryk-FLAG and Vangl2-HA constructs were co-transfected into cells, the levels of Vangl2 were greatly reduced in the membrane and increased in the cytoplasm in the presence of 200 ng ml^−1^ Wnt5a (Figure 5g, quantified in Figure 5h). In contrast, the change in localization of Vangl2 from the membrane to the cytoplasm was not detected in the co-transfected cells in the presence of 100 ng ml^−1^ Wnt5a (Figure 5g, quantified in Figure 5h). These results indicated that only Ryk was translocated to the cytoplasm in response to high concentration of Wnt5a. Moreover, in response to higher Wnt5a concentration, cytoplasmic distribution of Vangl2 might be facilitated by cytoplasmic translocation of Ryk through their physical interactions.

### Ryk translocation into the cytoplasm and Ryk–Vangl2 interaction are required for Vangl2 translocation into the cytoplasm

**Figure 6:**
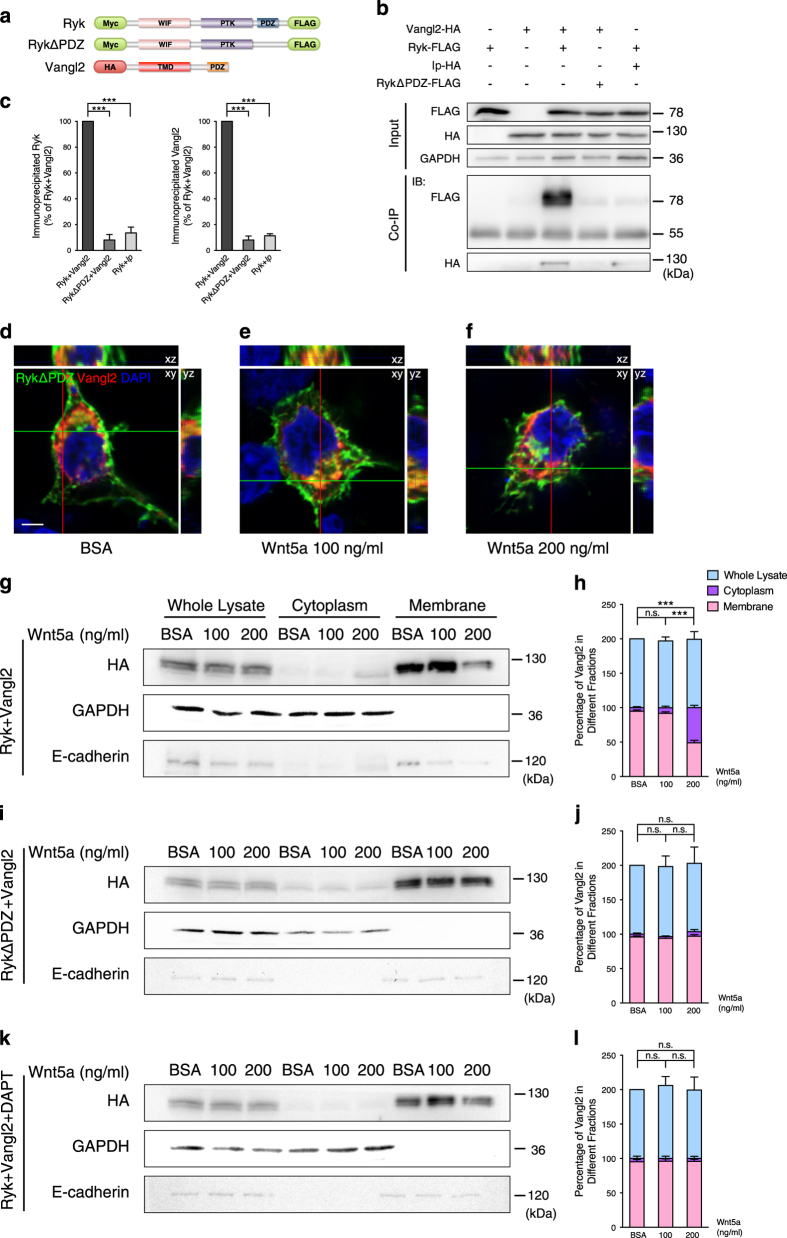
Disruption of Ryk translocation leads to reduction in cytoplasmic distribution of Vangl2. (**a**) Schematic diagram of the Ryk-FLAG and RykΔPDZ-FLAG constructs used in co-immunoprecipitation experiments. (**b**) Co-immunoprecipitation (Co-IP) of Ryk and Vangl2 from lysates of HEK293T cells transfected with Ryk, Vanlg2, Ryk and Vangl2, RykΔPDZ and Vangl2, or Ryk and *lp*. (**c**) Quantification of co-immunoprecipitation ratio in each group. The ratio was calculated and normalized to Ryk and Vangl2 group. Data are represented as the mean±s.e.m. of three independent experiments per group. ***P<0.001 (immunoprecipitated Ryk, *F*=209; immunoprecipitated Vangl2, *F*=672, one-way ANOVA). (**d**–**f**) Vangl2 distribution when co-expressed with RykΔPDZ. Vangl2-HA- and RykΔPDZ-FLAG-expressing cells were treated with BSA (**d**), 100 ng ml^−1^ wnt5a (**e**) or 200 ng ml^−1^ Wnt5a (**f**). DAPI staining (blue) labeled the nucleus. HA (red) and FLAG (green) staining show the expressed Vangl2 and RykΔPDZ, respectively. Scale bar, 5 μm. (**g**, **i**) Western blot analysis of whole-cell lysate, cytoplasm and membrane extracts from Vangl2-HA and Ryk-FLAG (**g**), or Vangl2-HA and RykΔPDZ-FLAG (**i**) co-expressing HEK293T cells. Cells were treated with BSA, 100 ng ml^−1^ Wnt5a or 200 ng ml^−1^ Wnt5a. (**k**) Western blot analysis of whole-cell lysate, cytoplasm and membrane extracts from Vangl2-HA and Ryk-FLAG co-expressing HEK293T cells treated with DAPT. Antibodies against GAPDH and E-cadherin were used to confirm the purity of the cytoplasm and the membrane fractions, respectively. (**h**, **j**, **l**) Quantification of the percentage of Vangl2 in the different fractions in (**g**, **i**, **k**), respectively (**h**, BSA and 200, 100 and 200 ng ml^−1^, *P*<0.0001;** j**, BSA and 100 ng ml^−1^, *P*=0.8402; BSA and 200 ng ml^−1^, *P*=0.7311; 100 and 200 ng ml^−1^, *P*=0.4239; **l**, BSA and 100 ng ml^−1^, *P*=0.9841; BSA and 200 ng ml^−1^, *P*=0.9920; 100 and 200 ng ml^−1^, *P*=0.9986). Data are represented as the mean±s.e.m. of at least three independent experiments per group. ***P<0.001, (one-way ANOVA). PTK, protein tyrosine kinase domain; NS, not significant; TMD, transmembrane domain; WIF, Wnt inhibitory factor motif.

To test the importance of Ryk–Vangl2 interaction in Vangl2 translocation into the cytoplasm, we made a Ryk mutant construct with the last four carboxyl-terminal residues, which make up the PDZ-binding domain, deleted [30] (RykΔPDZ; Figure 6a). As this domain is known to be responsible for binding to Vangl2, the RykΔPDZ protein should not be able to bind to Vangl2 [30]. RykΔPDZ disruption of Ryk and Vangl2 binding was first verified by co-immunoprecipitation. Ryk or RykΔPDZ was co-expressed in HEK293T cells with Vangl2. Subsequent co-immunoprecipitation showed that Ryk co-immunoprecipitated with Vangl2, and deletion of the Ryk PDZ domain greatly reduced the ability of Ryk to interact with Vangl2 (Figure 6b). Vangl2 *lp* mutant, which has an S464N amino acid substitution that impairs the ability of Vangl2 to physically interact with Dvl and Ror2 [32, 33], also failed to co-immunoprecipitate with Ryk (Figure 6b, quantified in Figure 6c). To further test the effect of RykΔPDZ, the RykΔPDZ-FLAG and Vangl2-HA constructs were then co-transfected into HEK293T cells in the presence of BSA, 100 or 200 ng ml^−1^ Wnt5a. In all three groups, co-localization of RykΔPDZ and Vangl2 was weakly detected in both the membrane and cytoplasm (Figure 6d–f), in contrast to the obvious co-localization of Ryk and Vangl2 in both the membrane and cytoplasm (Figure 5b–d).

Western blotting was carried out to confirm these results. In contrast to the substantial Vangl2 increase in the cytoplasm and decrease in the membrane of cells co-expressing Ryk and Vangl2 with 200 ng ml^−1^ Wnt5a (Figure 6g, quantified in Figure 6h), there was no detectable Wnt5a-induced change in Vangl2 localization in cells co-expressing RykΔPDZ and Vangl2 (Figure 6i, quantified in Figure 6j). These results strongly indicated that cytoplasmic distribution of Vangl2 in response to high concentration of Wnt5a depends on the interaction between Ryk and Vangl2. Previous studies have shown that Ryk is cleaved by γ-secretase and that the γ-secretase inhibitor DAPT can effectively reduce Ryk ICD levels through blocking Ryk cleavage [31]. In our experiments, DAPT addition efficiently disrupted the Vangl2 increase in the cytoplasm and decrease in the membrane in Ryk and Vangl2 co-expressing cells induced by 200 ng ml^−1^ Wnt5a (Figure 6k, quantified in Figure 6l). Taken together, these findings showed that Ryk–Vangl2 interaction and Ryk cleavage are crucial for cytoplasmic distribution of Vangl2 in response to high concentration of Wnt5a.

### Ryk and Vangl2 undergo asymmetric distribution to the cytoplasm in growth cones of corticospinal axons in response to Wnt5a gradient

**Figure 7:**
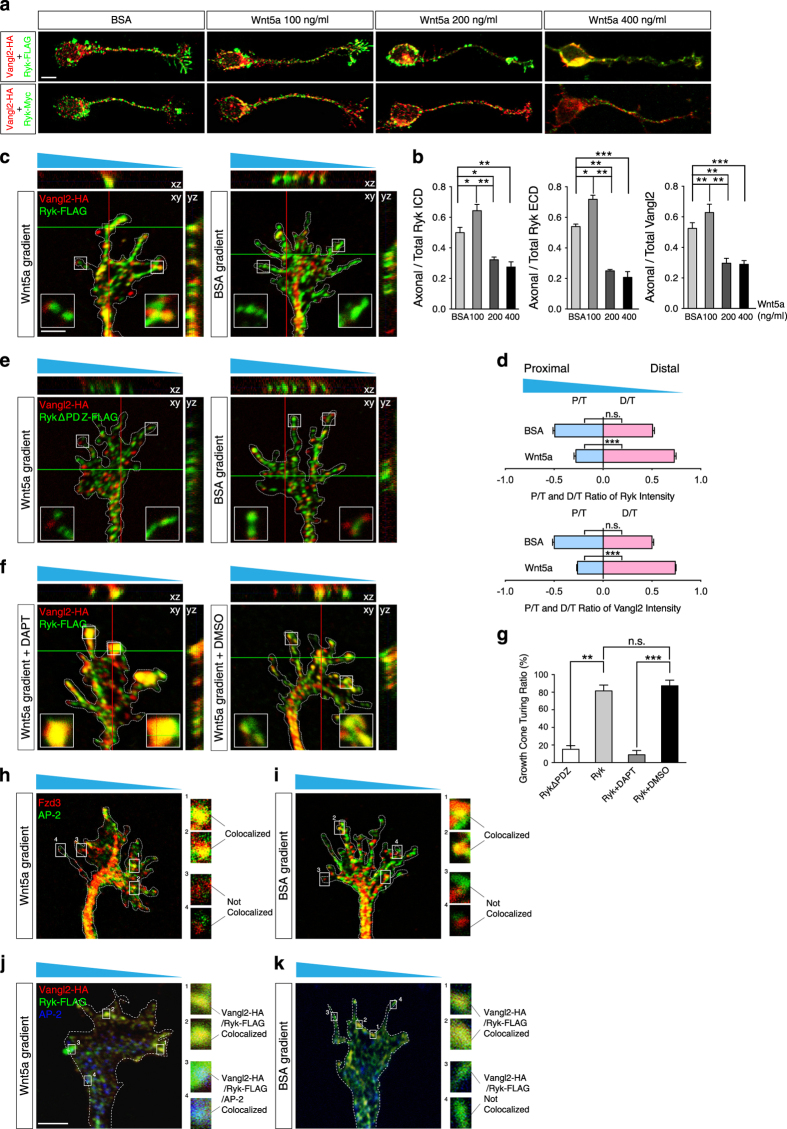
Cytoplasmic translocation of Ryk and Vangl2 are asymmetrically in growth cones of corticospinal axons under Wnt5a gradient. (**a**) Ryk and Vangl2 transport into corticospinal neurons in response to Wnt5a. E18.5 corticospinal neurons were co-transfected with Vangl2-HA and Myc-Ryk-FLAG. HA (red), Myc (green) or FLAG (green) antibodies were applied to label Vangl2-HA, Ryk ECD or Ryk ICD, respectively. Scale bar, 5 μm. (**b**) Quantification of expression ratios of Ryk ICD, Ryk ECD and Vangl2 in neurons. The expression intensity of Ryk ICD, Ryk ECD or Vangl2 in axons was measured and normalized to expression intensity of Ryk ICD, Ryk ECD or Vangl2 in entire neurons. Data are represented as the mean±s.e.m. of four independent experiments, each of which included at least 30 neurons per group. *P<0.05, **P<0.01, ***P<0.001 (Left, BSA and 100 ng ml^−1^, *P*=0.0480; BSA and 200 ng ml^−1^, *P*=0.0202; 100 and 200 ng ml^−1^, *P*=0.0011; BSA and 400 ng ml^−1^, *P*<0.0001; middle, BSA and 100 ng ml^−1^, *P*=0.0013; BSA and 200 ng ml^−1^, *P*<0.0001; 100 and 200 ng ml^−1^, *P*<0.0001; BSA and 400 ng ml^−1^, *P*<0.0001; right, BSA and 100 ng ml^−1^, *P*=0.0019; BSA and 200 ng ml^−1^, *P*<0.0001; 100 and 200 ng ml^−1^, *P*<0.0001; BSA and 400 ng ml^−1^, *P*<0.0001. One-way ANOVA). (**c**) Response of growth cones of corticospinal neurons co-transfected with Ryk and Vangl2 to Wnt5a and BSA gradients. Growth cones perpendicular to the gradient were selected, and divided into proximal and distal sides relative to the gradient. Immunostaining with HA (red) and FLAG (green) antibodies labeled Vangl2 and Ryk in the growth cones. (**d**) Quantification of the proximal/total (*P*/*T*) and distal/total (*D*/*T*) ratio of Ryk and Vangl2 intensity. Data are represented as the mean±s.e.m. of four independent experiments, with each experiment including at least 18 growth cones per group. ***P<0.001 (BSA, *P*=0.9653; Wnt5a, *P*<0.0001) and Vangl2 (**d**; BSA, *P*=0.9998; Wnt5a, *P*<0.0001. One-way ANOVA). (**e**) Response of growth cones of corticospinal neurons transfected with RykΔPDZ and Vangl2 to Wnt5a and BSA gradients. Immunostaining with HA (red) and FLAG (green) antibodies labeled Vangl2 and RykΔPDZ in the growth cones, respectively. (**f**) Response of Ryk- and Vangl2-expressing growth cones of dissociated corticospinal neurons to Wnt5a with either DAPT or DMSO treatment (1 μm). Immunostaining with HA (red) and FLAG (green) antibodies labeled Vangl2 and Ryk in the growth cones, respectively. (**g**) Quantification of growth cone-turning ratio in different groups. Data are represented as the mean±s.e.m. of three independent experiments. **P<0.01, ***P<0.001 (Ryk=81.4±6.56%, RykΔPDZ=15.1±4.19%, *P*=0.001. Ryk+DMSO=87.3±6.39%, Ryk+DAPT=8.9±4.85%, *P*<0.0001. One-way ANOVA). Each independent experiment included at least 20 neurons per group. (**h**, **i**) Response of growth cones of corticospinal neurons to Wnt5a or BSA gradient when transfected with Fzd3-FLAG. Immunostaining with FLAG (red) and α-adaptin (AP-2; green) antibodies labeled intracellular Fzd3 and α-adaptin, respectively. (**j**, **k**) Response of growth cones of corticospinal neurons to Wnt5a or BSA gradient when transfected with Vangl2-HA and Ryk-FLAG. Immunostaining with HA (green), FLAG (red) and α-adaptin (AP-2; blue) antibodies labeled Vangl2, intracellular Ryk and α-adaptin, respectively. The insets show the high-magnification images of boxed areas in (**c**, **e**, **f**, **h**–**k**), respectively. Scale bar, 2 μm (**c**, **e**, **f**, **h**–**k**). NS, not significant.

To track Ryk transfer in corticospinal neurons under Wnt5a gradient, we made a double-tagged Ryk expression construct, in which Ryk was tagged with Myc at the extracellular N terminus and with FLAG at the intracellular C terminus (Figure 6a). We co-expressed Myc-Ryk-FLAG and Vangl2-HA in corticospinal neurons in the presence of BSA or 100 or 200 or 400 ng ml^−1^ Wnt5a. In the BSA control, Ryk and Vangl2 co-localized in both axons and growth cones. In 100 ng ml^−1^ Wnt5a, the co-localization of Ryk and Vangl2 substantially increased in growth cones of corticospinal neurons. In 200 ng ml^−1^ Wnt5a, co-localization of Ryk ICD and Vangl2 in somas was obviously increased, whereas co-localization of Ryk ECD (extracellular domain) and Vangl2 became barely detectable in somas and axons of corticospinal neurons. In 400 ng ml^−1^ Wnt5a, co-localization of Ryk ICD and Vangl2 in somas was further increased, whereas co-localization of Ryk ECD and Vangl2 became barely detectable in somas and axons of corticospinal neurons (Figure 7a). Quantification of the percentages of Ryk ICD (Figure 7b, left), Ryk ECD (Figure 7b, middle) and Vangl2 (Figure 7b, right) in axonal portion of the corticospinal neurons also confirmed that Ryk and Vangl2 increased in axons in the presence of higher concentrations (100 and 200 ng ml^−1^) of Wnt5a.

To test whether the cytoplasmic translocation of Ryk and Vangl2 mediates growth cone turning, cultured corticospinal neurons were transfected with Ryk-FLAG and Vangl2-HA, and then exposed to a gradient of Wnt5a. In the control BSA gradient, Ryk and Vangl2 were equally distributed in the proximal and distal sides of the cells (relative to the BSA source), suggesting that Ryk and Vangl2 are expressed randomly in the absence of Wnt5a gradient. In contrast, there was a significant bias for distal distribution of Ryk and Vangl2 in the growth cones that were turned toward the lower Wnt5a concentration (Figure 7c). Quantification suggested that Ryk (Figure 7d, upper) and Vangl2 (Figure 7d, lower) were translocated to the cytoplasm more frequently exposed to higher Wnt5a levels and were maintained interacting in the membrane on the distal side of the growth cones exposed to lower Wnt5a levels.

RykΔPDZ was expressed in corticospinal neurons to disrupt the interactions between Ryk and Vangl2. In RykΔPDZ and Vangl2-expressing growth cones of corticospinal neurons, Ryk and Vangl2 co-localization was barely detectible in both the Wnt5a and BSA gradients, and the growth cones showed no obvious turning (Figure 7e). The quantification of the turning ratio of RykΔPDZ and Vangl2-expressing growth cones compared with Ryk and Vangl2-expressing growth cones also supported our observations (Figure 7g). These results demonstrated that disruption of Ryk–Vangl2 interaction efficiently abolishes growth cone turning in the Wnt5a gradient. When DAPT was added to block γ-secretase and interrupt Ryk cleavage in Ryk and Vangl2-expressing neurons, Ryk and Vangl2 co-localized in the tips of the growth cones symmetrically, and the growth cones did not show obvious turning in response to the Wnt5a gradient (Figure 7f). In contrast, in the DMSO vehicle control group, Ryk and Vangl2 co-localized asymmetrically, with most of the co-localization in the portion of the growth cones exposed to lower levels of Wnt5a. Concurrently, the growth cones turned to the lower concentration of Wnt5a (Figure 7f). The quantification of turning ratios in the DMSO and DAPT groups also supported our observations (Figure 7g). These results suggested that Wnt5a gradient leads to interactions and asymmetric distribution of Ryk and Vangl2 in growth cones, which is essential for growth cone turning away from the higher Wnt5a concentration.

We also examined Fzd3 localization in growth cone of cultured corticospinal neuron responding to a Wnt5a gradient. Fzd3-FLAG-expressing corticospinal neurons were exposed to a gradient of Wnt5a or BSA. Thirty minutes after the addition of Wnt5a or BSA, these growth cones were fixed, and immunostained with α-adaptin (AP-2 adaptor complex) [19] and FLAG antibodies. Fzd3 and AP-2 co-localization in axonal tips was primarily distributed on the side of lower Wnt5a levels (Figure 7h). In contrast, in the BSA gradient, Fzd3 and AP-2 co-localization in axonal tips was equally distributed on both sides (Figure 7i). These results suggested that Fzd3 is translocated to the cytoplasm more frequently on the side of lower Wnt5a levels. And similarly, we examined Vangl2 and Ryk co-localization in growth cone of cultured corticospinal neuron transfected with Ryk-FLAG and Vangl2-HA responding to a Wnt5a gradient. We found Vangl2, Ryk and AP-2 co-localization in axonal tips was primarily distributed on the side of lower Wnt5a levels (Figure 7j). In contrast, in the BSA gradient, Vangl2 and Ryk co-localization in axonal tips was equally distributed on both sides, but there is no co-localization with AP-2 (Figure 7k). These results suggested that Ryk and Vangl2 were translocated to the cytoplasm more frequently on the side of higher Wnt5a levels and were maintained interacting in the membrane on the distal side exposed to lower Wnt5a levels.

In summary, in low concentrations of Wnt5a, Ryk and Vangl2 interact and are maintained in the membrane. As a result, Fzd3 is translocated to the cytoplasm and activates downstream signaling. In high concentrations of Wnt5a, Ryk is cleaved and translocated to the cytoplasm and facilitates the cytoplasmic distribution of Vangl2, which inhibits cytoplasmic distribution of Fzd3 and results in the inhibition of downstream signaling.

### Synchronous translocation into the cytoplasm of Ryk and Vangl2 is required for Wnt5a repulsion of CST in the spinal cord

**Figure 8:**
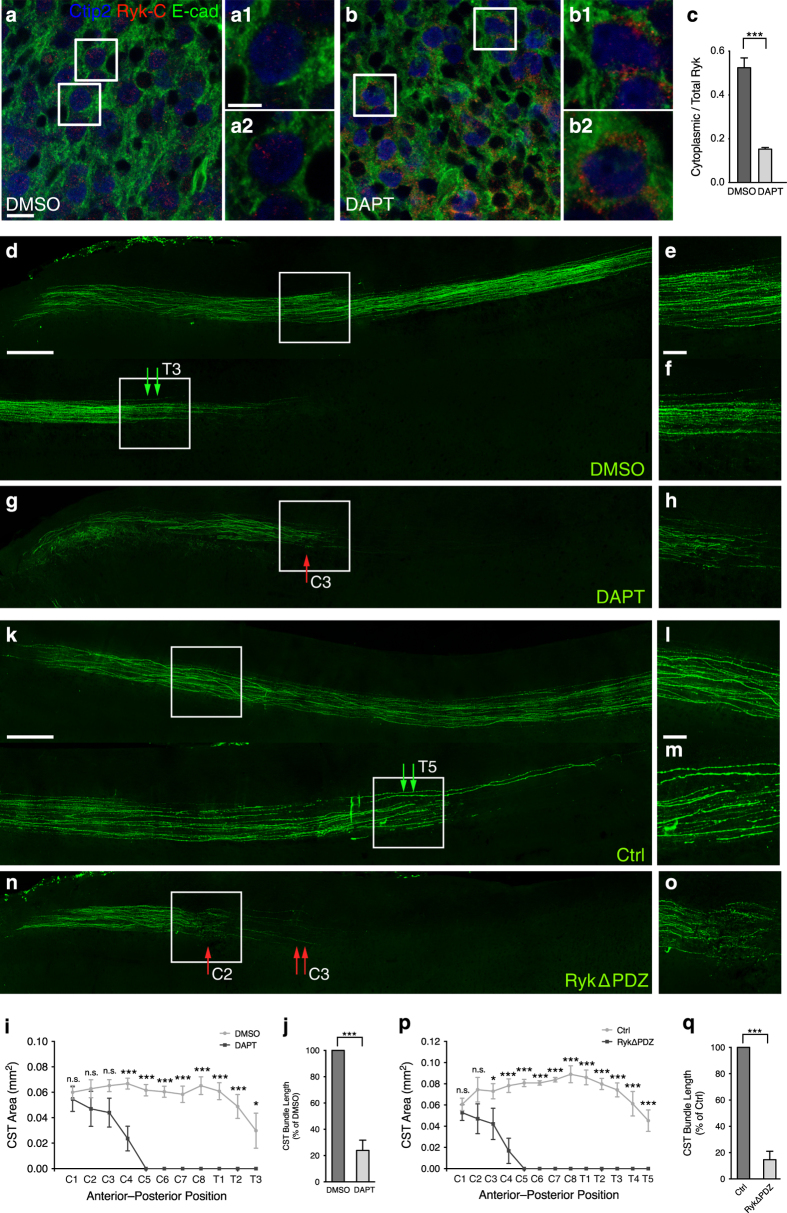
Synchronous translocation of Ryk and Vangl2 to the cytoplasm is required for Wnt repulsion of CST in the spinal cord. (**a**, **b**) The expression pattern of Ryk in coronal sections of cortex tissues injected with DMSO or DAPT. Anti-Ryk labeled Ryk ICD (red), anti-Ctip2 labeled corticospinal neurons (blue) and anti-E-cadherin (E-cad) labeled the cell membrane (green). Scale bar, 20 μm. (**a1**, **a2**, **b1**, **b2**) Higher magnification images of the boxed areas in **a**, **b**. Scale bar, 5 μm. (**c**) Quantification of the ratio of cytoplasmic Ryk to total Ryk. The expression intensity of Ryk in the cytoplasm was normalized to the expression intensity of Ryk in the entire neuron. Data are represented as the mean±s.e.m. of data from 10 mice in each group. At least 15 neurons were measured in each mouse. ***P<0.001 (DAPT=0.1520±0.0076%, DMSO=0.5235±0.0452%, *t*=8.100, *P*<0.0001. Student’s *t-*test). (**d**–**h**) CST axonal pathfinding in spinal cord of mice with cortex injection of DMSO or DAPT visualized with EGFP immunostaining. CST axons in sagittal sections of spinal cord with DMSO (**d**) or DAPT (**g**) injection. The double green arrows indicate control EGFP-expressing CST continuing to descend at the third thoracic (T3) segment. The red arrow indicates where DAPT-treated CST aberrantly terminates at the third cervical (C3) segment. (**e**, **f**, **h**) Higher magnification images of the boxed areas in **d**, **g**, respectively. Scale bar, 500 or 100 μm (higher magnification images). (**i**, **j**) Quantification of CST area and CST bundle length in each group. Data are represented as the mean±s.e.m. *P<0.05, ***P<0.001. Data of CST bundle length and CST area were analyzed from eight mice in each group, using two-way ANOVA (**i**, C1, *P>*0.9999; C2, *P*=0.7363; C3, *P*=0.3244; C4, *P*=0.0004; C5–T2, *P*<0.0001; T3, *P*=0.0369) or Student’s *t-*test (**j**, 100% of DMSO, DAPT=23.9±7.80, *t*=9.75, *P*<0.0001), respectively. (**k**–**o**) CST axonal pathfinding in spinal cord of mice with control lentivirus or RykΔPDZ-overexpression lentivirus injected into the cortex. CST axons were identified with EGFP expression (green). The double green arrows indicate control EGFP-expressing CST continuing to descend at the fifth thoracic (T5) segment. The red arrow indicates where RykΔPDZ-expressing CST present random extension at the second cervical (C2) segment. The double red arrow indicates where RykΔPDZ-expressing CST aberrantly ends at the third cervical (C3) segment. (**l**, **m**, **o**) Higher magnification images of the boxed areas in **k**, **n**, respectively. Scale bar, 500 or 100 μm (higher magnification images). (**p**, **q**) Quantification of CST area and CST bundle length in each group. Data are represented as the mean±s.e.m. *P<0.05, ***P<0.001. Data of CST area and CST bundle length were analyzed from five mice in each group, using two-way ANOVA (**p**, C1, *P*=0.9995; C2, *P*=0.0939; C3, *P*=0.0357; C4–T4, *P*<0.0001; T5, *P*=0.0002) or Student’s *t-*test (**q**, 100% of control, RykΔPDZ=14.7±6.41, *t*=13.3, *P*<0.0001), respectively.

*In vitro* analysis showed that either blocking Ryk cleavage with DAPT or interrupting Ryk–Vangl2 interactions with RykΔPDZ overexpression led to interruption of cytoplasmic Vangl2 distribution. We next examined the effect of DAPT and RykΔPDZ *in vivo*. We found that Ryk was in both the membrane and cytoplasm in the cortex injected with DMSO (Figure 8a,a1,a2). Comparatively, Ryk was mostly in the cell membrane in cortex injected with DAPT (Figure 8b,b1,b2). The ratio of cytoplasmic Ryk to total Ryk was calculated and quantified in each group. Quantification showed that ~60% of Ryk was in the cytoplasm in cortex injected with DMSO, whereas cytoplasmic Ryk was downregulated to ~16% with DAPT injection (Figure 8c). No statistically significant difference was detected in the number of corticospinal neurons between the DMSO- and DAPT-treated groups (data not shown). The Ryk-C antibody that recognizes the Ryk ICD was applied to detect the cytoplasmic distribution of Ryk in cortical layer 5 with DAPT or DMSO treatment. The result of Ryk ICD detection confirmed that the cytoplasmic distribution of Ryk was significant interrupted with cortical injection of DAPT (Supplementary Figure S3a and b). These result suggested that DAPT efficiently interrupts the translocation of Ryk into the cytoplasm and reduces the Ryk ICD levels in neonatal corticospinal neurons *in vivo*.

DMSO or DAPT was injected into P0 sensorimotor cortex with EGFP-expressing lentivirus *in vivo*, and the mice were killed 2 weeks after injection. We then used anti-EGFP staining of sagittal sections of spinal cord to analyze the pathfinding of spinal CST axons (Figure 8d–h). CST axons continued to extend over the third thoracic (T3) segment at P14 and presented normal pathfinding in DMSO-treated mice (Figure 8d–f, double green arrow in Figure 8d). CST axons presented random extension and aberrantly terminated at the third cervical (C3) segment in DAPT-treated mice (Figure 8g and h, red arrow in Figure 8g). The distance that CST axons descended into the spinal cord was substantially reduced in DAPT-treated mice (Figure 8g) compared with DMSO-treated mice (Figure 8d). Moreover, the size of CST-axon bundles in DAPT-treated mice (Figure 8h) also appeared much smaller than in DMSO-treated mice (Figure 8e). The area and length of the spinal CST in each group were measured and quantified. The quantification confirmed the significant reductions both in CST areas (Figure 8i) and CST length (Figure 8j) in the DAPT-treated group compared with the DMSO group. These *in vivo* observations strongly suggest that cytoplasmic translocation of Ryk is required for the pathfinding of CST axons in the spinal cord.

We next injected a RykΔPDZ-overexpression lentivirus or control EGFP-expressing lentivirus into corticospinal neurons of P0 mice and the mice were then kept alive for 2 weeks (Figure 8k–o). The control EGFP-expressing CST axons continued to extend over the fifth thoracic (T5) segment at P14 and presented normal pathfinding in the entire spinal cord (Figure 8k–m, double green arrow in Figure 8k). RykΔPDZ-expressing CST axons presented random extension at the second cervical (C2) segment (Figure 8n, red arrow) and aberrantly terminated at the third (C3) segment (Figure 8n, double red arrow). The area and distance of CST-axon descent into the spinal cord were substantially reduced in cortical RykΔPDZ-expressing mice compared with the control mice (Figure 8k–o). Quantification of CST areas (Figure 8p) and CST length (Figure 8q) also verified these observations. These findings strongly suggested that the interaction and cytoplasmic translocation of Ryk and Vangl2 are required for the pathfinding of CST axons, and this interaction relies on the Ryk PDZ domain.

To verify the specific interruption of Wnt/PCP pathway with cortical injection of DAPT and RykΔPDZ-expressing lentivirus, cortical injection of the Rho A-expressing lentivirus was applied to rescue the axon pathfinding errors of DAPT-treated or RykΔPDZ-expressing CST axons. The diagram showed the location of cortical injection (Supplementary Figure S4a). These mice were then killed 2 weeks post injection, and coronal sections of cortex or sagittal sections of spinal cord were taken for analysis. The coronal sections of cortex showed control EGFP-expressing and mCherry-expressing CST neurons (Supplementary Figure S4b,b1,d,d1,d2), EGFP-expressing and Rho A-expressing CST neurons (Supplementary Figure S4c,c1,c2) and RykΔPDZ-expressing and Rho A-expressing CST neurons (Supplementary Figure S4e,e1,e2). The sagittal sections of spinal cord showed DMSO-treated or control EGFP-expressing CST axons co-injected with mCherry-expressing lentivirus continued to extend over the fifth thoracic (T5) segment at P14 (Supplementary Figure S4f,l) and presented normal pathfinding in the entire spinal cord (Supplementary Figure S4g,h,m and n). And DAPT-treated or RykΔPDZ-expressing CST axons co-injected with Rho A-expressing lentivirus continued to extend over the second thoracic (T2) (Supplementary Figure S4i) or seventh cervical (C7) segment at P14 (Supplementary Figure S4o) and presented normal pathfinding in the spinal cord (Supplementary Figure S4j,k,p and q). Quantification of CST bundle length showed no significance between DMSO-treated CST axons with co-injection of Cherry-expressing lentivirus and DAPT-treated CST axons with co-injection of Rho A-expressing lentivirus or between control EGFP-expressing CST axons with co-injection of Cherry-expressing lentivirus and RykΔPDZ-expressing CST axons with co-injection of Rho A-expressing lentivirus (Supplementary Figure S4r and s). These results strongly indicate that Ryk mediated Wnt5a repulsion of CST axons via modulating PCP pathway.

Recent studies on the biochemical interactions of the core PCP pathway components have suggested a possibly general mechanism for setting up and maintaining the asymmetric localization of PCP pathway components in which Vangl2 antagonizes Dvl1-mediated Fzd3 inactivation by promoting cytoplasmic translocation of Fzd3 [20]. Our study proposes a working model to explain Ryk-mediated Wnt/PCP repulsion of CST axons in which low concentrations of Wnt5a allow Ryk and Vangl2 interaction and maintenance in the membrane, and this lead to Vangl2 inhibition of Dvl1. As a result, Fzd3 is translocated to the cytoplasm and activates downstream signaling (Supplementary Figure S5). In high concentrations of Wnt5a, Ryk is cleaved and translocated to the cytoplasm and facilitates the cytoplasmic translocation of Vangl2, which leads to release of Dvl1 and further inhibition of Fzd3, and finally results in the inhibition of downstream signaling (Supplementary Figure S5). Thus, this asymmetric PCP signal transduction mediated by Ryk interaction and translocation with Vangl2 ultimately leads to CST growth cone turning toward lower concentration of Wnt5a.

## Discussion

Recent studies have established the role of PCP signaling in Wnt-mediated axon guidance. The PCP pathway components Fzd3, Ceslr3 and Vangl2 were directly tested, and found to be required in A–P guidance of brain stem serotonergic and dopaminergic axons and the spinal cord commissural axons [18, 20]. In all of these studies, PCP seems to be the primary mediator of Wnt attraction of axonal growth cones. However, growth cones of corticospinal axons are also repelled by Wnt5a via Ryk [12, 27], and frizzled has also been reported to be involved in axon repulsion [15]. This raises an interesting question of whether Wnt attraction and repulsion of axons are mediated by distinct Wnt signal pathways or there is a common core mechanism of signal transduction underlying both Wnt attraction and repulsion of axons. Here, we found that Ryk regulates the PCP signaling pathway through bidirectional modulation of Vangl2 distribution to mediate Wnt repulsion of CST axons. Our findings elucidated the molecular mechanism underlying the cross-talk between Ryk and the PCP pathway, and showed that Ryk switches the PCP-mediated CST growth cone attraction to repulsion through modulating the asymmetric distribution of Vangl2. This finding also explains the molecular mechanism underlying bidirectional axonal guidance of the Wnt/PCP signal along the A–P axis.

Two Wnt signaling pathways have been characterized: the canonical and non-canonical Wnt pathways [34, 35]. The canonical Wnt signaling pathway is transduced via the Wnt/GSK3ß signaling pathway, and the non-canonical Wnt signaling pathway is transduced via the Wnt/PCP signaling pathway, which controls tissue polarity and cell movement. In both the canonical and non-canonical Wnt signaling pathways, Ryk has been identified as a receptor of Wnt. The Wnt/Ryk signaling pathway plays an essential role in the establishment of major axon tracts in the developing nervous system [36]. Our results in this study connect the PCP signaling pathway with Ryk, and we found that Ryk mediates the PCP signaling pathway in the development of the CST. However, considering that there are six core components of the PCP signaling pathway, it is too early to conclude that our findings show all aspects of the cross-talk between Ryk and PCP signaling. We also cannot exclude the possibility that intracellular molecules downstream in the pathways are involved in the cross-talk between Wnt/Ryk and Wnt/PCP signal pathways. Previous studies have shown that Wnt/calcium pathway is thought to involve downstream RhoGTPases [37]. This Wnt signaling pathway could ultimately regulate growth cone behaviors by targeting cytoskeletal proteins [38], and calcium signaling is mediated by Ryk [15]. These findings suggest that more than one class of Wnt receptors mediates calcium signaling. But there is no result linking specific receptor types to distinct calcium signaling pathways, and there exists possibility that Ryk mediates the cross-talk between Wnt/ calcium pathway and Wnt/PCP pathway. Additional studies need to be carried out to address these questions and to further explore the potential networks involved in the intracellular signal transduction of Wnt/PCP signaling pathway.

Axons are guided along different routes anteriorly to posteriorly by attractive or repulsive cues in the extracellular environment. Previous studies have identified some highly conserved families of guidance molecules involved in this process, including Wnts, Shh, semaphorins, Slits and netrins. These guidance molecules steer axons by regulating cytoskeletal dynamics in the growth cone through signaling pathways. These guidance cues are interrelated and their cross-talk is important in axon guidance. In our shRNA injection experiments, a small percentage of the transfected axons displayed normal A–P trajectory. This may be due to incomplete knockdown of target proteins by shRNA [39] and/or other unidentified signal pathways being involved in regulation of axon pathfinding. Although many A–P axon guidance molecules have been identified, the mechanisms underlying their cross-talk are still far from completely understood. The ultimate goal is to understand the interactions between these guidance molecules and mechanisms underlying their cross-talk. Many more signal molecules undoubtedly still await discovery.

## Materials and methods

### Animals and treatments

All mice were maintained and handled according to the guidelines and regulations of the Animal Research Committee of Soochow University. CD-1 mice were obtained from SLAC Inc (Shanghai, China).

### Plasmids, inhibitors and antibodies

Mouse Myc-Ryk was provided by Dr Helen Cooper [27] (The University of Queensland and FLAG sequences were inserted into the C terminus. The final four Ryk residues were deleted to produce Ryk∆PDZ [30]. Vangl2 and Vangl2 *looptail* mutant (*lp*) with N-terminal HA tag were provided by Dr Yingzi Yang [32] (Harvard School of Dental Medicine). Fzd3 was provided by Dr Yimin Zou [19] (University of California, San Diego). All constructs were verified by sequencing. Recombinant Wnt5a was purchased from R&D Systems. DAPT was purchased from Sigma-Aldrich (St Louis, MO, USA). The antibodies used in this study include anti-Ryk (Abgent, San Diego, CA, USA), anti-Vangl2 (Santa Cruz Biotechnology, Dallas, TX, USA), anti-Fzd3 (Sigma-Aldrich), anti-Dvl1 (Santa Cruz Biotechnology), anti-Tuj1 (Abcam, Cambridge, UK), anti-FLAG (Sigma-Aldrich), anti-HA (Sigma-Aldrich), anti-Myc (Cell Signaling Technology, Danvers, MA, USA), anti-α-Adaptin (BD Biosciences, Franklin Lakes, NJ, USA), anti-E-cadherin (BD Biosciences), anti-Ctip2 (Abcam) and anti-GAPDH (Sigma-Aldrich). Dylight 488-conjugated phalloidin and Alexa Fluor-conjugated secondary antibodies for mouse/rabbit/rat/goat IgG and mouse IgM were purchased from Invitrogen (Waltham, MA, USA).

### shRNA and lentivirus constructs

Sequences of the shRNA constructs were as follows: mouse Ryk shRNA (5′- TGAAAGATGGTTACCGAATATTCAAGAGATATTCGGTAACCATCTTTCTTTTTT-3′), mouse Vangl2 shRNA (5′- GGGAGAAACAACAACGGTG-3′), mouse Fzd3 shRNA (5′- CCGGCCTAATCTTCTGAACCATTCTCAAGAGAAATGGTTCAGAAGATTAGGTTTTTTG-3′) and mouse Dvl1 shRNA (5′- TTGAATCTAGCAGCTTTAT-3′) were purchased from Genechem Co., Ltd (Shanghai, China). The shRNA constructs were packed into lentivirus, using the hU6-MCS-Ubiquitin-EGFP vector, by Genechem Co., Ltd. The titrations of lentivirus for infection were as follows: 2×10^8^ TU ml^−1^ (Ctrl shRNA), 2.2×10^9^ TU ml^−1^ (Ryk shRNA), 1.05×10^8^ TU ml^−1^ (Vangl2 shRNA), 1.4×10^8^ TU ml^−1^ (Fzd3 shRNA) and 1.5×10^8^ TU ml^−1^ (Dvl1 shRNA). Sequences of the Rho A constructs were as follows: mouse Rho A (5′- GAGGATCCCCGGGTACCGGTCGCCACCATGGCTGCCATCAGGAAG-3′) was overexpressed by lentivirus and was purchased from Genechem Co., Ltd. The Rho A constructs were packed into lentivirus, using the Ubi-MCS-3FLAG-SV40-Cherry vector, by Genechem Co., Ltd. The titrations of lentivirus for infection were as follows: 2×10^8^ TU ml^−1^ (Cherry), 2.2×10^9^ TU ml^−1^ (Rho A).

### Immunohistochemistry

Mouse tissues were fixed in 4% paraformaldehyde (PFA) overnight at 4 °C. After equilibration with 30% (w/v) sucrose in PBS, the fixed tissues were embedded in OCT compound (Sakura, Tokyo, Japan) and frozen. For cortex immunohistochemistry, 20 μm thick sections were prepared, washed in PBS and then incubated in PHT (blocking solution) for 30 min at room temperature. Slides were further incubated for 24 h at 4 °C with rabbit anti-Ryk (1:100; Abgent), goat anti-Vangl2 (1:100; Santa Cruz Biotechnology), rabbit anti-Fzd3 (1:100; Sigma-Aldrich), mouse anti-Dvl1 (1:50; Santa Cruz Biotechnology), mouse anti-E-cadherin (1:1000; BD Biosciences) and rat anti-Ctip2 (1:500; Abcam) diluted in blocking solution. The slides were washed in PBS, then incubated 12 h at 4 °C with Alexa Fluor-conjugated secondary antibodies (Invitrogen, Thermo Fisher, Waltham, MA, USA; 1:1000) diluted in blocking solution, washed again and mounted using Fluoromount-G (SouthernBiotech, Birmingham, AL, USA). For cultured cell immunohistochemistry, cells were fixed with 4% PFA for 15 min at room temperature, blocked using PHT for 30 min at room temperature, incubated at 4 °C overnight with rabbit anti-Ryk (1:200), goat anti-Vangl2 (1:200), rabbit anti-Fzd3 (1:200), mouse anti-Dvl1 (1:200), mouse anti-Tuj1 (1:500; Abcam), mouse anti-FLAG (1:500; Sigma-Aldrich), rabbit anti-HA (1:500; Sigma-Aldrich), mouse anti-Myc (1:500; Cell Signaling Technology), and rat anti-Ctip2 (1:500) diluted in blocking solution and then incubated with diluted secondary antibodies (1:1000; Invitrogen) at room temperature for 1 h. Images were taken using a Zeiss (Oberkochen, Germany) confocal microscope.

### Corticospinal neuron culture

Cerebral cortices were dissected from embryonic day 18.5 (E18.5) mice in Leibovitz’s L-15 Medium (Invitrogen). Corticospinal neurons in layer 5 of the cerebral cortex were dissociated and plated at a density of 1×10^5^ per well on coverslips (Fisher Scientific, Waltham, MA, USA) coated with 10 μg ml^−1^ poly-d-lysine in 24-well dishes and cultured in Neurobasal medium (Invitrogen) containing 1× B27 supplement, 0.5% penicillin/streptomycin and 0.5 mm glutamine. After 24 h of culture in Neurobasal media, the cells were fixed with 4% PFA at room temperature for 15 min and immunostained. Confocal images were taken with a Zeiss microscope. To determine the efficiency of Vangl2, Fzd3 and Dvl1 shRNA-lentivirus knockdown, corticospinal neurons infected with lentivirus were seeded at 1×10^6^ per well in six-well dishes coated with poly-d-lysine and cultured for 72 h in Neurobasal medium. The cells were then lysed on ice and the lysates were analyzed by western blotting.

### Wnt5a gradient in Dunn chamber

The chamber consists of two concentric annular wells ground into one face of the slide (referred to as the inner and outer wells) separated by an ~1 mm wide bridge precisely 20 μm lower than the slide. Thus, when the chamber is covered with a coverslip carrying neurons, there is a gap between coverslip and bridge of 20 μm (Figure 2c). When the inner well is filled with control medium and the outer well with medium containing a protein, a linear diffusion gradient is quickly established in this gap and can be maintained for several hours [40]. For Dunn chamber assays, primary corticospinal neurons were dissociated and electroporated with constructs and then seeded on poly-d-lysine-coated coverslips (Fisher Scientific) at a low density, (1×10^5^ per well in a 24-well dish) such that individual isolated neurons were present and grown for 24 h. For DAPT-blocking experiments, at 6 h post electroporation, neurons were treated with 1 μm DAPT or DMSO for 18 h. The Dunn chamber (Hawksley DCC100) was prewashed twice with Neurobasal medium. Initially, both wells were filled with Neurobasal medium and the coverslip seeded with neurons was inverted onto the chamber in an offset position to leave a filling slit. The coverslip was sealed around the edges using hot Vaseline petroleum jelly except for the filling slit. The Neurobasal medium was then removed from the outer well and replaced with Neurobasal medium containing Wnt5a (200 ng ml^−1^). The slit was then sealed and the chamber was put back into the 37 °C incubator. Under these conditions, the gradient is known to become linear within ~20 min and have a half-life of ~24 h [41]. After 30 min, the coverslip was quickly removed from the Dunn chamber and put into 4% PFA for 15 min at room temperature for fixation followed by immunostaining. To quantify Ryk/Vangl2 expression, only the growth cones that were directly perpendicular to the Wnt5a gradient were analyzed. A line was drawn down the center of the growth cone image and the intensity of Ryk/Vangl2 expression in the proximal (P) and distal (D) sides were measured manually. The proximal/total (*P*/*T*) and distal/total (*D*/*T*) ratios were then calculated. To quantify the growth cone-turning ratio, turning angel was defined as the angel between midline and the final position of the growth cone (Figure 2c). Growth cone turning >30° was counted as turning growth cone. The number of turning growth cones was counted and normalized to the number of total growth cones responding to a gradient.

### Cell transfection and subcellular fractionation

HEK293T cells were cultured in DMEM (Gibco, Grand Island, NY, USA) supplemented with 10% fetal bovine serum and 1% penicillin/streptomycin. Transfections were carried out using Vigofect (Vigorous Biotechnology, Beijing, China). Forty-eight hour after transfection, HEK293T cells were incubated on ice for 30 min in cold sucrose HEPES buffer (320 mm sucrose, 20 mm HEPES, 5 mm EDTA) with PMSF (Amresco, Solon, OH, USA), phosphatase inhibitors (Roche, Basel, Switzerland) and protease inhibitors (Roche). The homogenate was centrifuged 15 min at 1000 *g*, and the supernatant was centrifuged again at 100 000* g* for 1 h to obtain the membrane (pellet) and the cytosolic (supernatant) fractions. The membrane pellets were resuspended in lysis buffer containing 1% Triton X-100 (v/v) for 30 min on ice, and the insoluble fractions were discarded. The total extracts and subcellular fractions were analyzed by western blotting.

### Co-immunoprecipitation and western blotting

Extracts from E18.5 and postnatal day 0 (P0) mice or transfected cells were incubated on ice for 30 min in cold RIPA buffer (50 mm Tris-HCl, 150 mm NaCl, 1 mm EDTA, 1% NP-40) with PMSF, phosphatase inhibitors and protease inhibitors. Afterwards the lysates were preincubated with Ryk or Vangl2 antibody for 1 h. Then antibodies were immobilized on Protein A/G agarose beads (Santa Cruz Biotechnology) overnight at 4 °C. The beads were precipitated by centrifugation at 6 000 r.p.m. for 1 min, and immunoprecipitates were washed three times in RIPA buffer and subjected to western blotting analysis. The protein samples were separated by 10% SDS-PAGE and transferred onto PVDF membranes. After blocking in blocking buffer (0.5% milk) for 1 h at room temperature, the blots were incubated with rabbit anti-Ryk (1:500), goat anti-Vangl2 (1:500), rabbit anti-Fzd3 (1:500), mouse anti-Dvl1 (1:200), rabbit anti-GAPDH (1:1000; Sigma-Aldrich), mouse anti-E-cadherin (1:1000), mouse anti-FLAG (1:1000) or rabbit anti-HA (1:1000), and subsequently with a peroxidase-conjugated secondary antibody.

### *In vivo* analysis

To express shRNA or Ryk∆PDZ in CST axons, 0.6 μl of shRNA-EGFP-expressing lentivirus or Ryk∆PDZ-EGFP-expressing lentivirus was injected into the motor cortex at 0.8 mm from the midline, and the depth of the injection was 0.5 mm. Mice were kept alive for 2 weeks before killing, respectively. For DAPT or DMSO injection experiments, 1 μl of 1 mm DAPT or DMSO was injected into sensorimotor cortex of P0 mice with EGFP-expressing lentivirus, and the mice were kept alive for 2 weeks before killing, respectively.

To express Rho A and shRNA or Ryk∆PDZ in CST axons, 0.6 μl of Rho A-cherry-expressing lentivirus and shRNA or Ryk∆PDZ-EGFP-expressing lentivirus was injected into the motor cortex at 0.8 mm from the midline, and the depth of the injection was 0.5 mm. Mice were kept alive for 2 weeks before killing, respectively. For DAPT or DMSO injection experiments, 1 μl of 1 mm DAPT or DMSO was injected into sensorimotor cortex of P0 mice with EGFP- and cherry-expressing lentivirus, and the mice were kept alive for 2 weeks before killing, respectively.

All of the injection sites were determined by the bregma point. EGFP-expressing and Cherry-expressing CST axons were directly observed under confocal microscope. CST length and CST area in the spinal cord were measured using Image J (NIH Software, Bethesda, MD, USA) as described previously [12].

### Statistical analysis

Statistical analysis was performed using Prism 6 (GraphPad Software, San Diego, CA, USA). Data are presented as mean±s.e.m. Statistical analysis for multiple comparisons was performed using one-way analysis of variance or two-way analysis of variance, and for two group comparison was performed using Student’s *t*-test.
