# Supplementary material for: Ryk regulates Wnt5a repulsion of mouse corticospinal tract through modulating planar cell polarity signaling
Source: Cell Discov. 2017 May 16;3:17015–. doi: 10.1038/celldisc.2017.15 (PMC5475318; doi:10.1038/celldisc.2017.15)
Supplement: Supplementary information [file celldisc201715-s1.pdf]

Supplementary Figure 1

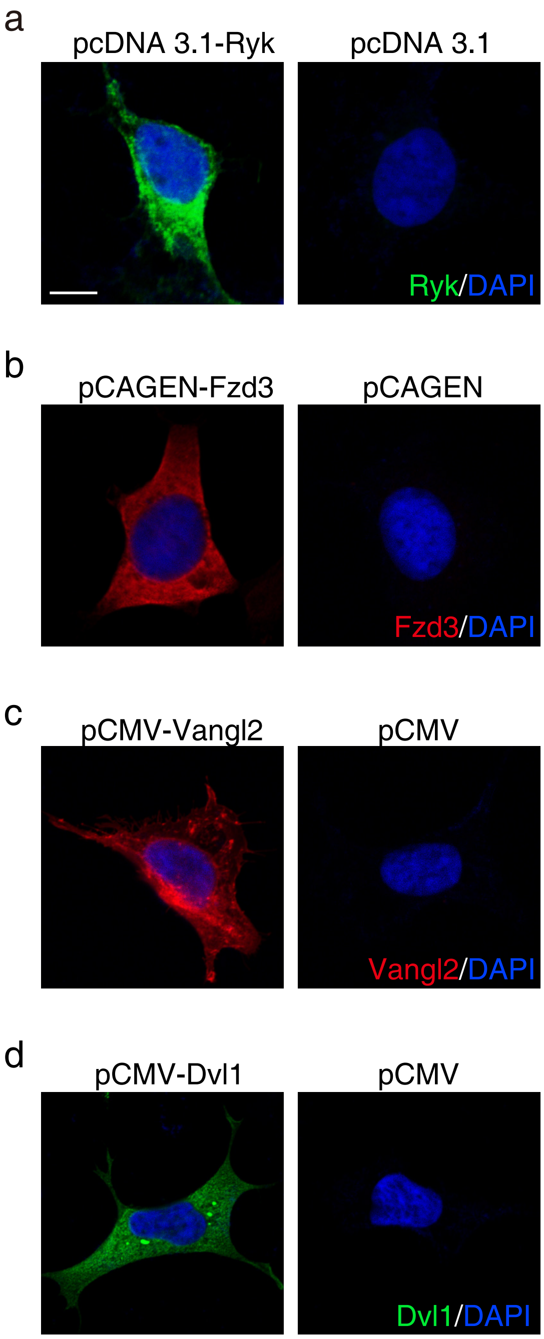

Supplementary Figure 2

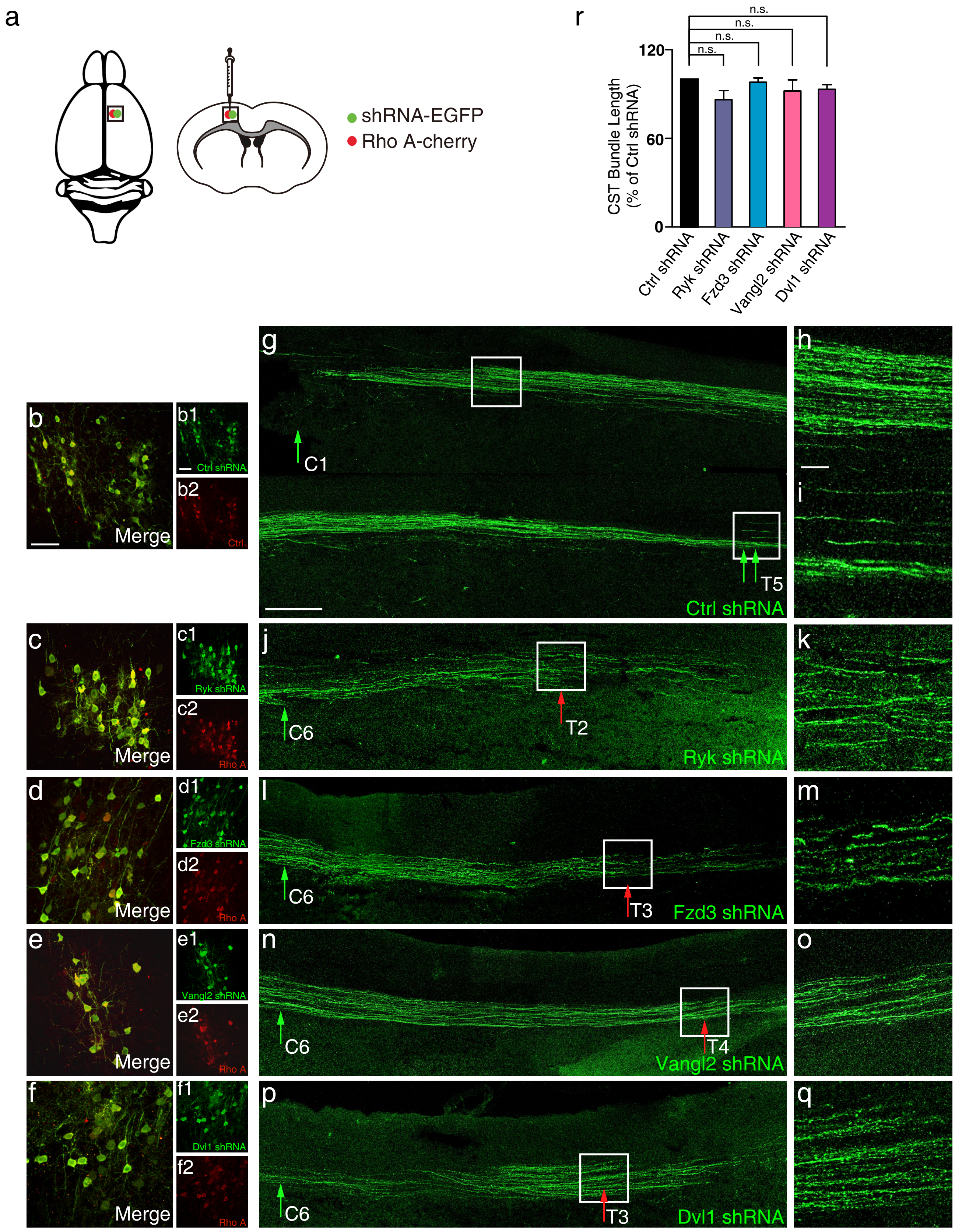

Supplementary Figure 3

a

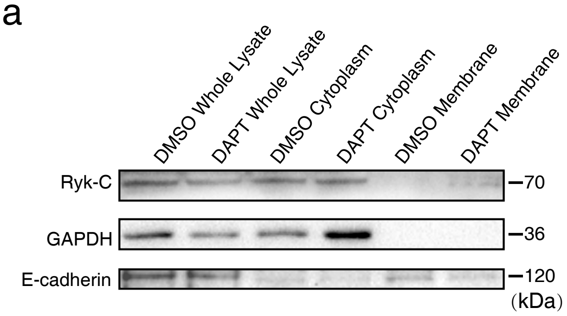

b

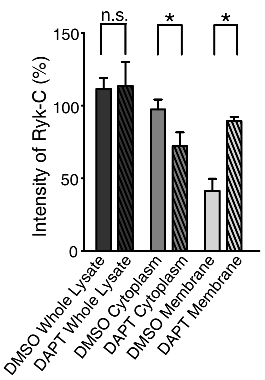

Supplementary Figure 4

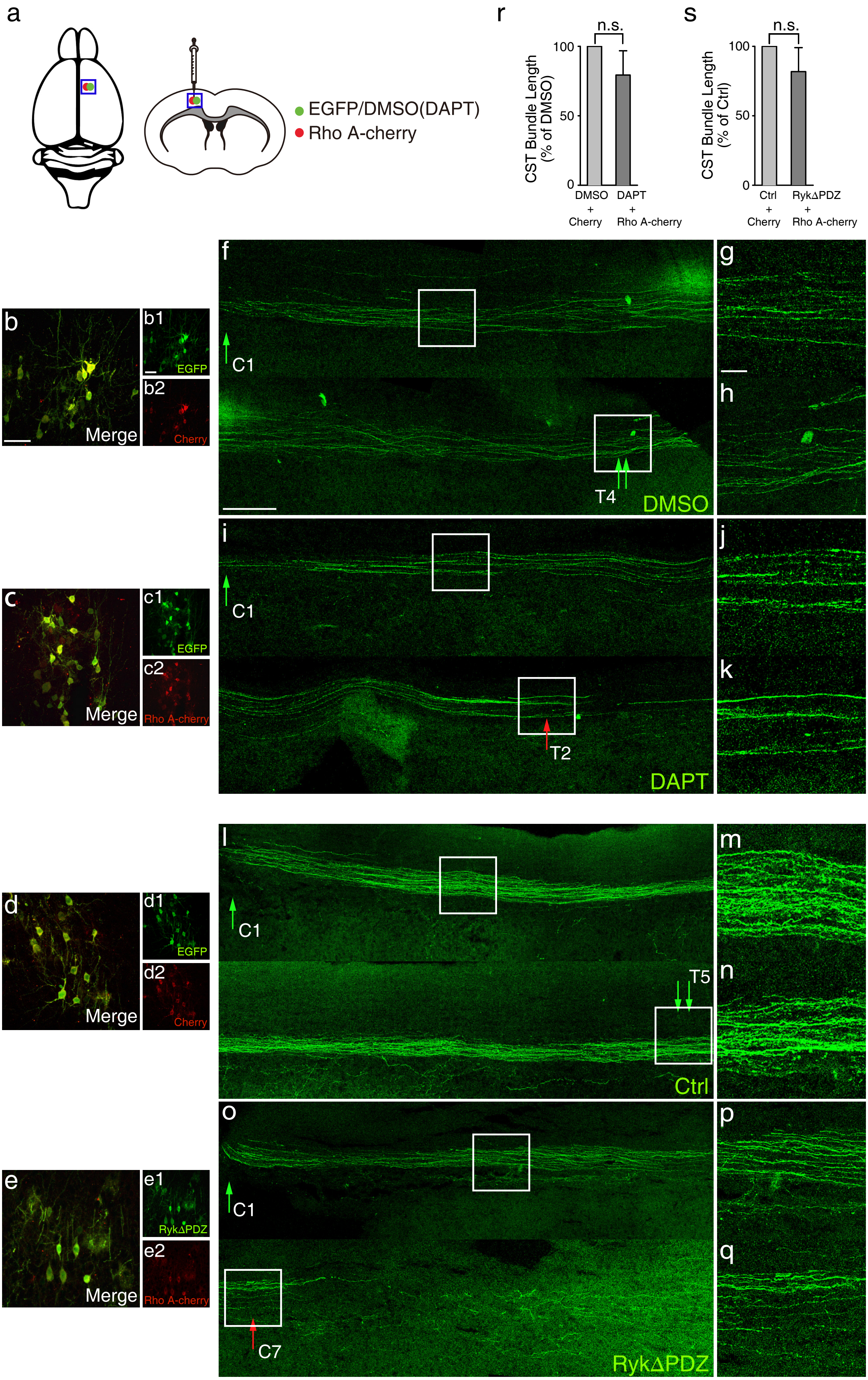

Supplementary Figure S5

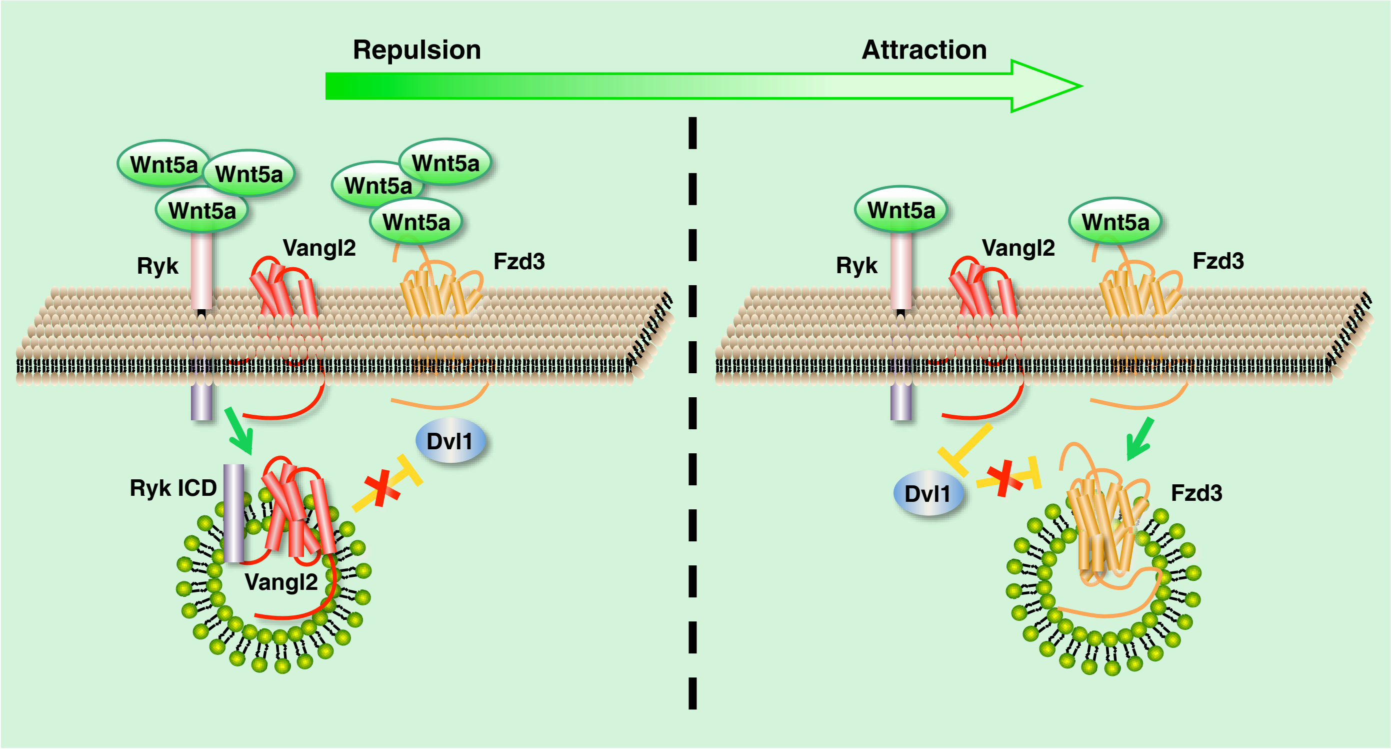

SUPPLEMENTARY FIGURE LEGENDS

Supplementary Figure S1 Negative controls of Ryk, Fzd3, Vangl2 and Dvl1 in HEK 293T cells. (**a**) HEK 293T cells transfected with pcDNA3.1-Ryk and pcDNA 3.1 were immunostained with Ryk (green) antibody and DAPI (blue). (**b**) HEK 293T cells transfected with pCAGEN-Fzd3 and pCAGEN were immunostained with Fzd3 (red) antibody and DAPI (blue). (**c**) HEK 293T cells transfected with pCMV-Vangl2 and pCMV were immunostained with Vangl2 (red) antibody and DAPI (blue) labeled. (**d**) HEK 293T cells transfected with pCMV-Dvl1 and pCMV were immunostained with Dvl1 (green) antibody and DAPI (blue) labeled.

Supplementary Figure S2 Rho A-expressing CST axons present normal pathfinding with knockdown of Ryk and PCP pathway components in corticospinal neurons. (**a**) The diagram of cortical co-injection with EGFP-expressing or shRNA EGFP-expressing (green) and Cherry-expressing or Rho A cherry-expressing (red) lentivirus. (**b, b1, b2**) Co-expression of control shRNA-EGFP (green) and control Cherry (red) in CST neurons. (**c, d, e, f**) Co-expression of Ryk, Fzd3, Vangl2, Dvl1 shRNA-EGFP (green) and Rho A-cherry (red) in CST neurons, respectively. (**c1, c2; d1, d2; e1, e2; f1, f2**) showed single channels in (**c, d, e, f**). (g) EGFP-expressing CST axons in developing spinal cord. The single green arrow indicates a CST projection initiation site in the spinal cord at 1st cervical (C1) segment. The double green arrows indicate control shRNA-expressing CST continuing to descend at the 5th thoracic (T5) segment. (**h, i**) Higher magnification images of the boxed areas in (**g**). (**j**) Ryk shRNA-expressing CST axons in developing spinal cord. (**k**) Higher magnification image of boxed area in (**j**). (**l**) Fzd3 shRNA-expressing CST axons in

developing spinal cord. (**m**) Higher magnification image of the boxed area in (**l**). (**n**) Vangl2 shRNA-expressing CST axons in developing spinal cord. (**o**) Higher magnification image of the boxed area in (**n**). (**p**) Dvl1 shRNA-expressing CST in developing spinal cord. (**q**) Higher magnification image of the boxed area in (**p**). (**r**) Quantification of CST bundle length in each shRNA-expressing group (% of control shRNA). Data are represented as the mean ± SEM. n.s. not significant. Data of CST bundle length was analyzed from at least seven mice in each group using one-way ANOVA respectively (100% of control shRNA, Ryk shRNA= 86.22 ± 6.182%, p = 0.1555; Fzd3 shRNA = 98.05 ± 2.846%, p = 0.5640; Vangl2 shRNA = 92.15 ± 7.437%, p = 0.4017; Dvl1 shRNA = 93.23 ± 3.127%, p = 0.1628). Scale bar, (**b, b1, b2; c, c1, c2; d, d1, d2; e, e1, e2; f, f1, f2**) 50μm, (**g, j, l, n, p**) 500 μm or 100 μm (higher magnification images).

Supplementary Figure S3 Western blot to detect Ryk ICD. (**a**) Western blot analysis to detect Ryk ICD of whole-cell lysate, cytoplasm and membrane extracts from cortex tissues injected with DMSO or DAPT. (**b**) Quantification of western blot analysis in (**a**). Data are represented as the mean ± SEM. *p < 0.05, n.s. not significant (DMSO whole-cell lysate = 111.6 ± 7.625%, DAPT whole-cell lysate = 113.7 ± 16.38%, t = 0.1651, p = 0.8841; DMSO cytoplasm = 97.41 ± 6.813%, DAPT cytoplasm = 72.35 ± 9.330%, t = 9.869, p = 0.0101; DMSO membrane = 41.45 ± 8.308%, DAPT membrane = 89.44 ± 2.828%, t = 6.095, p = 0.0259. Student's t-test).

Supplementary Figure S4 Rho A expression can rescue the pathfinding errors of DAPT treated or RykΔPDZ-expressing CST axons. (**a**) The diagram of cortical co-injection with EGFP-expressing or shRNA EGFP-expressing (green) and

Cherry-expressing or Rho A Cherry-expressing (red) lentivirus. (**b, b1, b2**) DMSO co-injected with EGFP-expressing and Cherry-expressing lentivirus in CST neurons. Co-expression of EGFP (green) and control Cherry (red) in CST neurons. (**c, c1, c2**) DAPT co-injected with EGFP-expressing and Rho A-expressing lentivirus in CST neurons. Co-expression of EGFP (green) and Rho A-Cherry (red) in CST neurons. (**d, d1, d2**) Co-expression RykΔPDZ-EGFP (green) and Cherry (red) in CST neurons. (**e, e1, e2**) Co-expression EGFP (green) and Rho A-Cherry (red) in CST neurons. (**f**) EGFP-expressing CST axons in developing spinal cord. The single green arrow indicates a CST projection initiation site in the spinal cord at 1st cervical (C1) segment. The double green arrows indicate control shRNA-expressing CST continuing to descend at the 5th thoracic (T5) segment. (**g, h**) Higher magnification images of the boxed areas in (**f**). (**i**) EGFP-expressing CST axons in developing spinal cord. The single green arrow indicates a CST projection initiation site in the spinal cord at 1st cervical (C1) segment. The double green arrows indicate control shRNA-expressing CST continuing to descend at the 2ed thoracic (T2) segment. (**j, k**) Higher magnification images of the boxed areas in (**i**). (**l**) EGFP-expressing CST axons in developing spinal cord. The single green arrow indicates a CST projection initiation site in the spinal cord at 1st cervical (C1) segment. The double green arrows indicate control shRNA-expressing CST continuing to descend at the 5th thoracic (T5) segment. (**m, n**) Higher magnification images of the boxed areas in (**l**). (**o**) RykΔPDZ -expressing CST axons in developing spinal cord. The single green arrow indicates a CST projection initiation site in the spinal cord at 1st cervical (C1) segment. The double green arrows indicate control shRNA-expressing CST continuing to descend at the 5th thoracic (T5) segment. (**p, q**) Higher magnification images of the boxed areas in (**o**). (**r, s**) Quantification of CST bundle length in each shRNA-expressing

group. Data are represented as the mean ± SEM. n.s. not significant. Data of CST bundle length were analyzed from five mice in each group, using Student's *t*-test (**r**, % of DMSO + Cherry, = 79.37 ± 10.09%, t=0.1776, p=2.044; s, % of control shRNA + Cherry, RykΔPDZ + Rho A Cherry = 81.83 ± 9.959%, t=1.824, p=0.2097) Scale bar, (**b, b1, b2; c, c1, c2; d, d1, d2; e, e1, e2**) 50μm, (**f, i, l, o**) 500 μm or 100 μm (higher magnification images).

Supplementary Figure S5 Schematic illustration of the Ryk–PCP cross-talk modulating Wnt repulsion of CST axons. A working model to explain the Ryk-mediated Wnt/PCP repulsion of CST axons. In high concentrations of Wnt5a, Ryk is cleaved and translocated to the cytoplasm and facilitates the cytoplasmic translocation of Vangl2, which leads to release of Dvl1 and inhibition of Fzd3 and finally to the inhibition of downstream signaling. In low concentrations of Wnt5a, Ryk and Vangl2 interact and are maintained in the membrane, and this lead to Vangl2 inhibition of Dvl1 and Fzd3 translocation, which activates downstream signaling. Thus the growth cones of corticospinal neurons extend towards the lower concentration of Wnt in response to a Wnt5a gradient.
